# MnO_2_ Nanowires with Sub‐10 nm Thick Conjugated Microporous Polymers as Synergistic Triboelectric Materials

**DOI:** 10.1002/advs.202409917

**Published:** 2024-11-03

**Authors:** Hanbyeol Jung, Dong‐Min Lee, Jina Park, Taeho Kim, Sang‐Woo Kim, Seung Uk Son

**Affiliations:** ^1^ Department of Chemistry Sungkyunkwan University Suwon 16419 South Korea; ^2^ Department of Materials Science and Engineering Yonsei University Seoul 03722 South Korea; ^3^ Center for Human‐Oriented Triboelectric Energy Harvesting Yonsei University Seoul 03722 South Korea

**Keywords:** conjugated microporous polymer, manganese dioxide, nanowire, triboelectric material, triboelectric nanogenerator

## Abstract

MnO_2_ nanowires coated with conjugated microporous polymers (CMP) are applied as triboelectric energy harvesting materials. The tribopositive performance of the CMP shells is enhanced with the assistance of MnO_2_ nanowires (MnO_2_ NW), likely due to cationic charge transfer from the tribopositive CMP layers to the surface Mn^2+^ and Mn^3+^ species of MnO_2_ NW. This is supported by model studies. The MnO_2_@CMP‐2 with sub‐10 nm thick CMP layers shows promising triboelectric output voltages up to 576 V and a maximum power density of 1.31 mW cm^−2^. Spring‐assisted triboelectric nanogenerators fabricated with MnO_2_@CMP‐2/PVP‐3 films are used as power supplies to operate electronic devices.

## Graphical abstract

MnO_2_@CMP nanowires show promising triboelectric performance. The possible cationic charge transfer from tribopositive CMP shells to the surface Mn^2+^/Mn^3+^ species of MnO_2_ nanowires is proposed as a mechanistic principle. TENGs fabricated with a MnO_2_@CMP‐2/PVP‐3 film show the output peak‐to‐peak voltage of 576 V and a maximum power density of 1.31 mW cm^−2^.

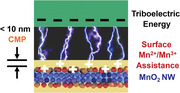

## 1 Introduction

Over the past decade, research on small‐scale energy harvesting and utilization has gained considerable significance.^[^ ^1^ ^]^ For instance, triboelectric energy in everyday life can be harvested and utilized for various purposes.^[^ ^2^ ^]^ Since the Wang group developed triboelectric nanogenerators (TENGs),^[^ ^3^ ^]^ extensive research has focused on fabricating new devices.^[^ ^4^ ^]^ In addition, to improve the efficiency of TENGs, extensive studies have been conducted on triboelectric materials.^[^ ^5^ ^]^

As triboelectric energy harvesting materials, various organic polymers have been studied.^[^ ^6^ ^]^ In addition, the surface areas and electronic properties of polymers have been tuned to enhance their triboelectric performance.^[^ ^7^, ^8^, ^9^ ^]^ For example, microporous organic materials with high surface areas have been studied for engineering TENGs.^[^ ^7^, ^8^ ^]^ As a class of microporous organic materials, conjugated microporous polymers (CMPs) have been prepared by the coupling of organic building blocks.^[^ ^10^ ^]^ Recently, our research group reported that CMPs can be used as promising tribopositive materials for harvesting triboelectric energy.^[^ ^11^ ^]^

On the other hand, porous organic polymer‐inorganic composites have been studied to achieve enhanced triboelectric performance.^[^ ^12^ ^]^ The triboelectrification performance of organic polymers can be enhanced with the assistance of inorganic nanomaterials. While organic polymer materials offer advantages in facile chemical engineering, inorganic nanomaterials can provide additional benefits such as facilitating redox reactions.

Recently, MnO_2_ nanomaterials have been utilized as energy storage materials in pseudocapacitors.^[^ ^13^ ^]^ The morphology of MnO_2_‐based nanomaterials has been engineered into nanowires. Usually, the surface of MnO_2_ nanowires has an amorphous character and consists of Mn^2+^ and Mn^3+^ species,^[^ ^14^ ^]^ which can be further oxidized to Mn^4+^ species by donating electrons.

It can be speculated that core–shell MnO_2_@CMP nanowires can be engineered to enhance the tribopositive performance of CMP materials with the assistance of surface Mn^2+^ and Mn^3+^ species in the inner MnO_2_ nanowires. When preparing MnO_2_@CMP nanowires, we observed the facile generation of static electricity in a Falcon plastic tube (Figure S1 and Movie S1, Supporting Information). In this work, we report the preparation of MnO_2_@CMP nanowires and their enhanced triboelectric performance, compared to the corresponding MnO_2_ and CMP materials.

## 2 Results and Discussion

**Figure 1:**
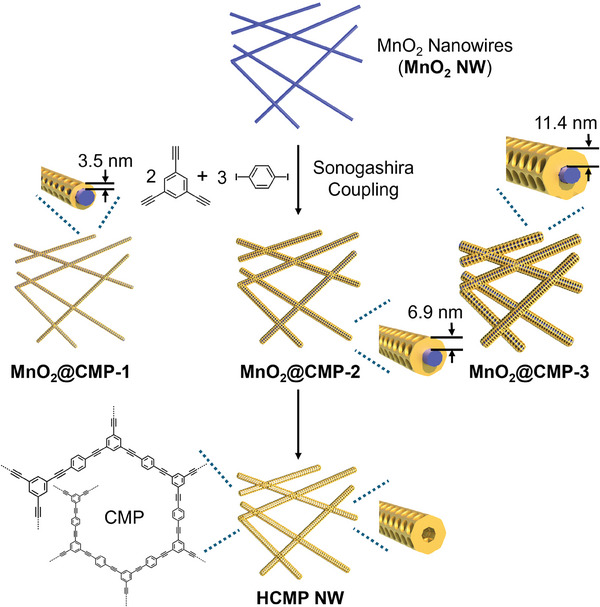
Synthesis of MnO_2_@CMP nanowires and HCMP nanowires (HCMP NW).

**Figure** **1** displays the synthetic scheme of MnO_2_@CMP nanowires.

First, MnO_2_ nanowires (MnO_2_ NW) were prepared by reacting KMnO_4_ with H_2_O_2_ in acetic acid.^[^ ^15^ ^]^ Through the Sonogashira coupling of 1,3,5‐triethynylbenzene with 1.5 eq. 1,4‐diiodobenzene in the presence of MnO_2_ NW, CMP layers were formed on the MnO_2_ NW. With a fixed amount of MnO_2_ NW (0.20 g), the amount of 1,3,5‐triethynylbenzene was gradually increased from 0.10 to 0.20 and 0.40 mmol to form three different MnO_2_@CMP nanowires (denoted as MnO_2_@CMP‐1, MnO_2_@CMP‐2, and MnO_2_@CMP‐3, respectively). As control materials, the inner MnO_2_ of MnO_2_@CMP‐2 was etched by treating with HCl to form hollow CMP nanowires (HCMP NW).

**Figure 2:**
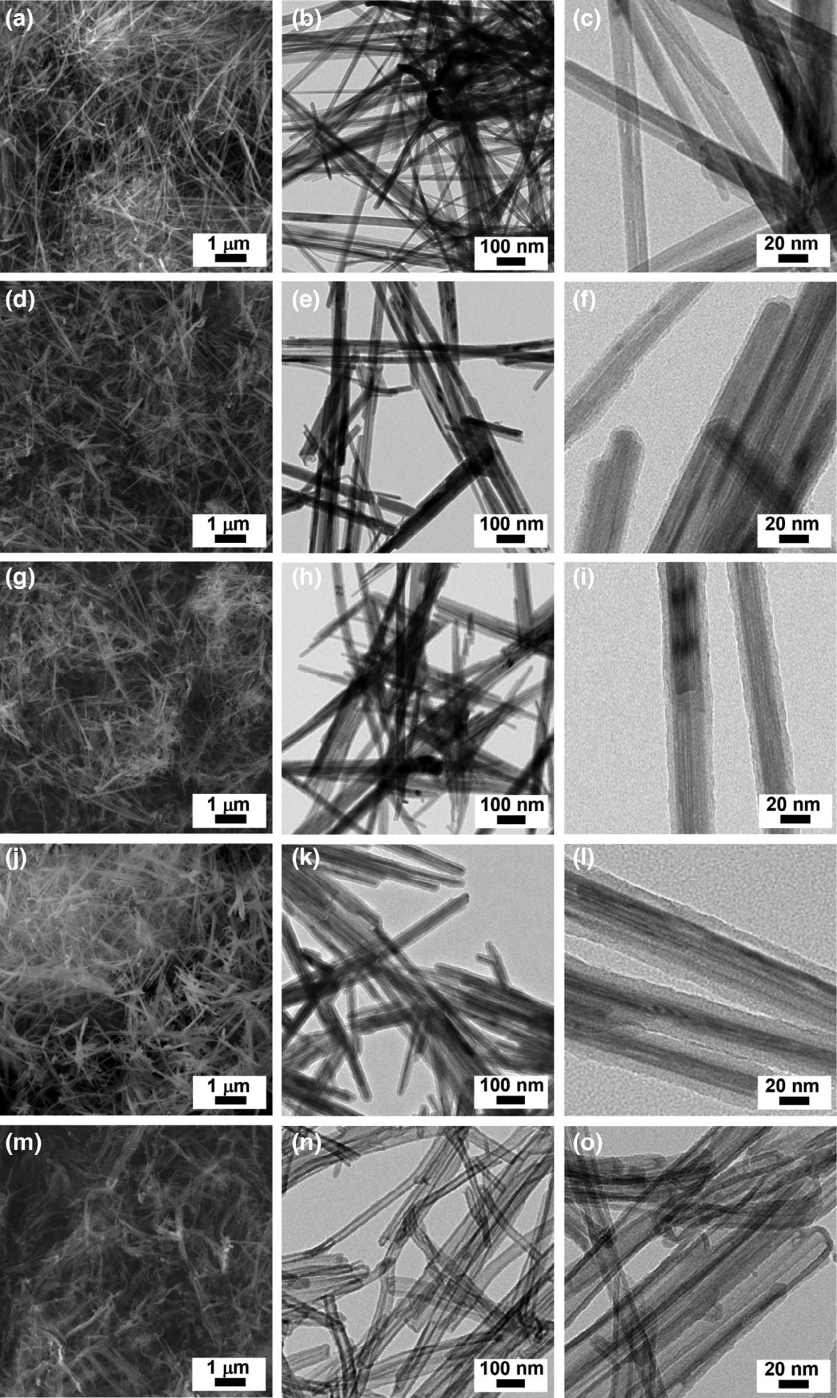
SEM and TEM images of (a,b,c) MnO_2_ NW, (d,e,f) MnO_2_@CMP‐1, (g,h,i) MnO_2_@CMP‐2, (j,k,l) MnO_2_@CMP‐3, and (m,n,o) HCMP NW.

The morphologies of materials were examined using scanning (SEM) and transmission electron microscopy (TEM) (**Figure** **2**). The SEM and TEM images of MnO_2_ NW revealed long nanowires with a thickness of 15–25 nm and a length of 2–5 µm (Figure 2a–c). While MnO_2_@CMPs retained wire‐like morphologies (Figure 2d,e,g,h,j,k), a closer inspection revealed a thin coating of CMP on the surface of MnO_2_ NW (Figure 2f,i,l). The coating thicknesses of CMPs in MnO_2_@CMP‐1, MnO_2_@CMP‐2, and MnO_2_@CMP‐3 were measured to be 3.5 ± 0.4, 6.9 ± 0.7, and 11.4 ± 0.7 nm, respectively (Figure S2, Supporting Information). Whilst the SEM images of HCMP NW showed wire‐like morphologies, the empty inner space could be confirmed by TEM analysis (Figure 2m–o).

**Figure 3:**
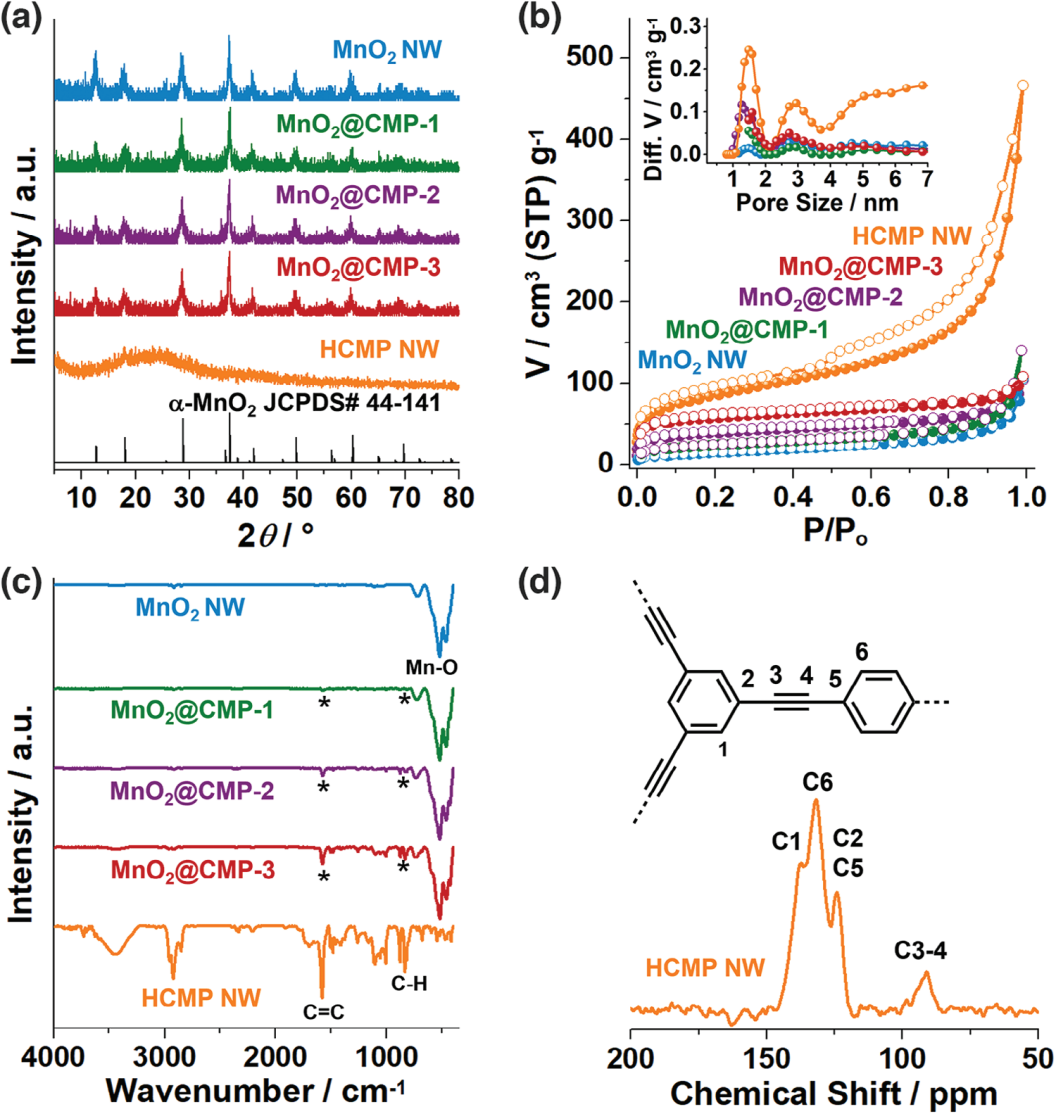
a) PXRD patterns, b) N_2_ adsorption–desorption isotherm curves obtained at 77 K and pore size distribution diagrams based on the NL‐DFT method, c) IR spectra of MnO_2_ NW, MnO_2_@CMPs, and HCMP NW. d) A solid‐state ^13^C NMR spectrum of HCMP NW.

The chemical and physical features of materials were investigated using various analytical techniques (**Figure** **3**). Powder X‐ray diffraction (PXRD) studies on MnO_2_ NW and MnO_2_@CMPs showed diffraction peaks at 12.5°, 17.9°, 28.6°, 37.5°, 41.6°, 50.0°, 60.3°, and 69.6°, corresponding to the (110), (200), (310), (211), (301), (411), (521), and (541) crystalline planes of α‐MnO_2_ (JCPDS# 44–141) (Figure 3a).^[^ ^16^ ^]^ In comparison, HCMP NW exhibited an amorphous feature, which is a conventional characteristic of CMPs in the literature.^[^ ^17^ ^]^

N_2_ sorption studies were conducted to investigate the surface areas and porosity of materials (Figure 3b). The analysis of N_2_ sorption isotherm curves based on the Brunauer–Emmett–Teller (BET) theory indicated that the MnO_2_ NW has a surface area of 54 m^2^ g^−1^ and poor porosity. In comparison, the HCMP NW showed a high surface area of 310 m^2^ g^−1^ and microporosity (pore sizes < 2 nm) with a total pore volume (V_t_) of 0.46 cm^3^ g^−1^. As the amount of CMPs increased in the MnO_2_@CMPs, the surface areas gradually increased from 85 m^2^ g^−1^ (MnO_2_@CMP‐1) to 138 (MnO_2_@CMP‐2) and 207 m^2^ g^−1^ (MnO_2_@CMP‐3) with the increase of V_t_ from 0.11 cm^3^ g^−1^ to 0.12 and 0.13 cm^3^ g^−1^, respectively.

The infrared (IR) absorption spectra of MnO_2_ NW and MnO_2_@CMPs revealed broad peaks at 460–520 cm^−1^, corresponding to Mn─O vibrations (Figure 3c).^[^ ^18^ ^]^ In comparison, HCMP NW showed aromatic C═C and C─H vibration peaks at 1581 and 837 cm^−1^, respectively, in addition to the C─O vibration at 1010–1105 cm^−1^, which was generated through the oxidation of CMP by MnO_2_ at the interfaces.^[^ ^19^ ^]^ As expected, as the amount of CMP increased in MnO_2_@CMPs, the corresponding vibration peaks of CMPs gradually increased (indicated by asterisks in Figure 3c). A solid‐state ^13^C nuclear magnetic resonance (NMR) spectrum of HCMP NW exhibited alkyne and aromatic carbon peaks at 91 and 124–138 ppm, respectively, consistent with the CMPs reported in the literature (Figure 3d).^[^ ^20^ ^]^

**Figure 4:**
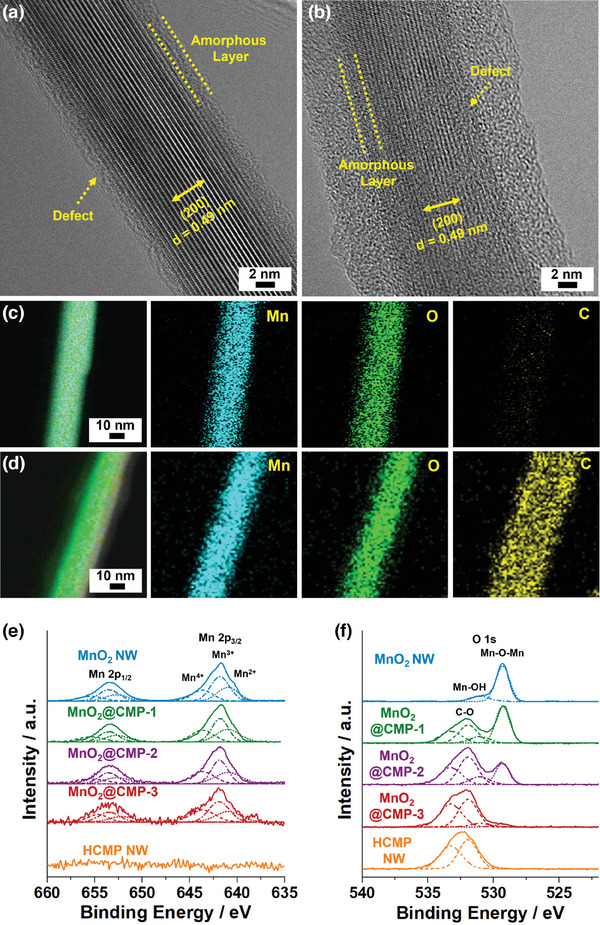
HR‐TEM images of a) MnO_2_ NW and b) MnO_2_@CMP‐2. EDS elemental mapping images of c) MnO_2_ NW and d) MnO_2_@CMP‐2. e,f) XPS Mn 2p and O 1s orbital peaks of MnO_2_ NW, MnO_2_@CMPs, and HCMP NW with normalized intensities (Refer to Figure S3, Supporting Information for unnormalized ones).

The high‐resolution (HR)‐TEM analysis of MnO_2_ NW and MnO_2_@CMP‐2 showed a crystalline inner part showing the (200) crystal plane of α‐MnO_2_ with an interlayer distance of 0.49 nm (**Figure** **4**a,b).^[^ ^21^ ^]^ The surface (a depth of ≈1.8 nm) of MnO_2_ NW displayed amorphous and defective features (indicated by dotted lines in Figure 4a,b). The MnO_2_@CMP‐2 showed the additional coating of amorphous CMP materials on the MnO_2_ NW (Figure 4b).

Energy dispersive X‐ray spectroscopy (EDS)‐based elemental mapping studies of MnO_2_ NW showed homogeneous distributions of Mn and O elements (Figure 4c). In comparison, MnO_2_@CMP‐2 showed a homogeneous distribution of carbon elements on the surface of MnO_2_ NW, indicating a successful coating of CMP (Figure 4d).

The chemical surroundings of materials were investigated by X‐ray photoelectron spectroscopy (XPS). The XPS Mn 2p_3/2_ and 2p_1/2_ orbital peaks of MnO_2_ NW and MnO_2_@CMPs were analyzed into three sets (Figure 4e; Figures S3 and S4, Supporting Information). Whilst the Mn 2p_3/2_ and 2p_1/2_ orbital peaks of Mn^2+^ species appeared at 641.0 and 652.7 eV, respectively, those of Mn^3+^ species appeared at 641.8 and 653.5 eV, respectively.^[^ ^22^ ^]^ In comparison, those of Mn^4+^ species were observed at 643.7 and 655.4 eV, respectively.^[^ ^22^ ^]^ The ratios of Mn^4+^/(Mn^2+^+Mn^3+^) of MnO_2_ NW, MnO_2_@CMP‐1, MnO_2_@CMP‐2, and MnO_2_@CMP‐3 were analyzed to be 0.31, 0.38, 0.42, and 0.44, respectively. These observations indicate that the surface of MnO_2_ NW consists of defective Mn^2+^ and Mn^3+^ species. The O 1s orbital spectrum of MnO_2_ NW showed two peaks at 529.3 and 530.3 eV, corresponding to the Mn─O─Mn and Mn─OH species, respectively (Figure 4f; Figures S3 and S5, Supporting Information).^[^ ^23^ ^]^ The O 1s orbital spectra of MnO_2_@CMPs and HCMP NW showed additional peaks at 531.9 and 533.2 eV, corresponding to C─O species that were generated through oxidation of CMP by MnO_2_ at the interfaces.^[^ ^24^ ^]^

**Figure 5:**
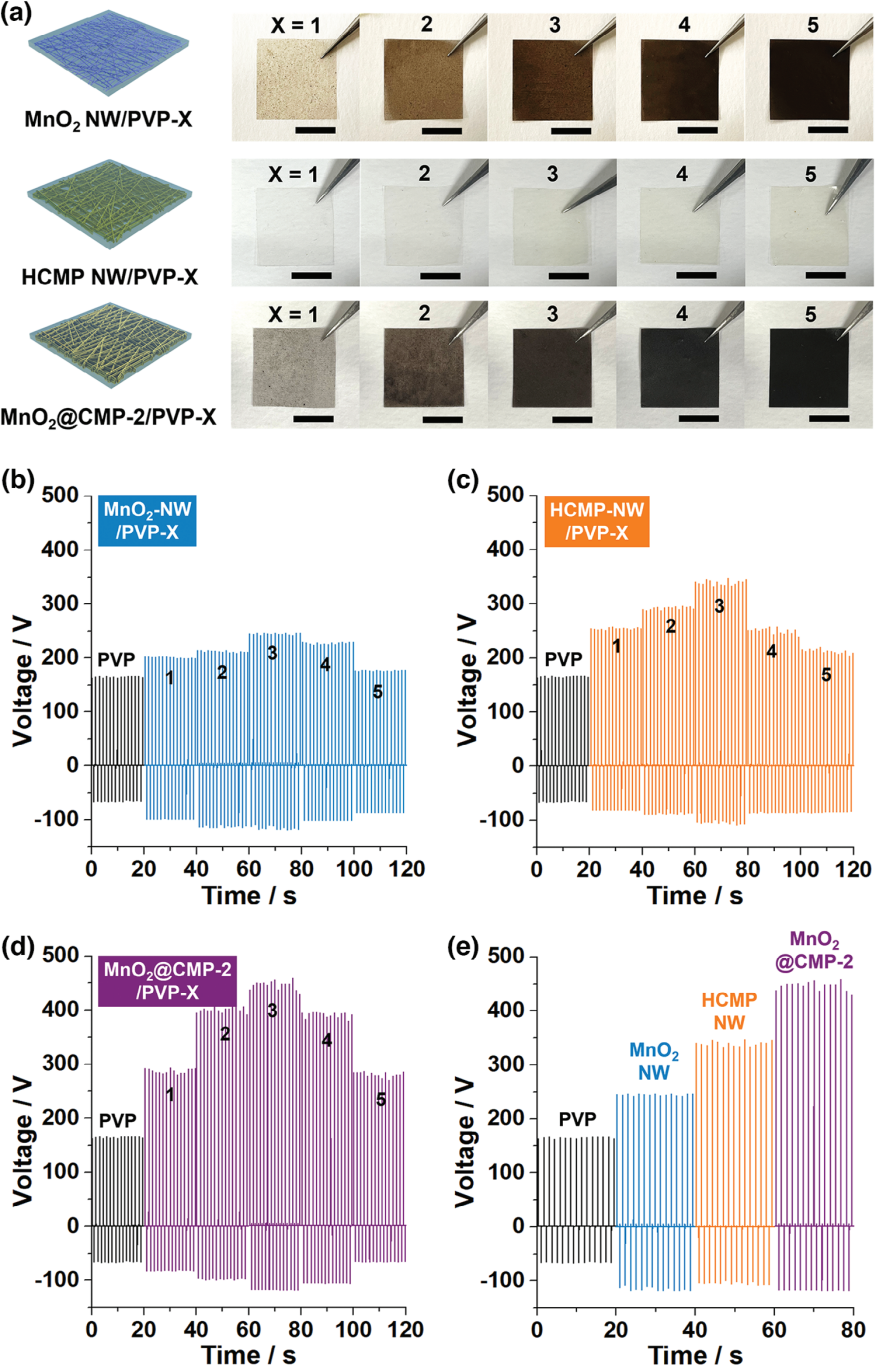
a) Photographs of MnO_2_ NW/PVP‐1–5, HCMP NW/PVP‐1–5, and MnO_2_@CMP‐2/PVP‐1–5 films (scale bars: 1 cm). Triboelectric output voltages of b) MnO_2_ NW/PVP‐1–5, c) HCMP NW/PVP‐1–5, d) MnO_2_@CMP‐2/PVP‐1–5 films. e) Comparative display of the output voltages of PVP, MnO_2_ NW/PVP‐3, MnO_2_@CMP‐2/PVP‐3, and HCMP NW/PVP‐3 films (a working area of 2 cm × 2 cm, pushing force of 2 kgf, pushing frequency of 0.73 Hz, RH 50%, PFA as a tribonegative material. Refer to Figure S11 (Supporting Information) for the output currents of corresponding films.

To study triboelectric performance, the films of materials were fabricated using polyvinylpyrrolidone (PVP) as a matrix (**Figure** **5**a and refer to Experimental Section in the Supporting Information). First, five MnO_2_ NW/PVP films containing 1, 3, 5, 7, and 10 wt.% MnO_2_ NW were fabricated (corresponding films were denoted as MnO_2_ NW/PVP‐1–5, respectively). As the amount of MnO_2_ NW increased, the brown color of the films became more intense (Figure 5a). According to thermogravimetric analysis (TGA), the content of MnO_2_ NW in MnO_2_@CMP‐2 was analyzed to be 72 wt.% (Figure S6, Supporting Information). Considering the information, five brown MnO_2_@CMP‐2/PVP films containing 1.4, 4.2, 6.9, 9.7, and 13.9 wt.% MnO_2_@CMP‐2 (corresponding to 1, 3, 5, 7, and 10 wt.% MnO_2_ NW and 0.4, 1.2, 1.9, 2.7, and 3.9 wt.% CMP‐2, respectively) were fabricated and denoted as MnO_2_@CMP‐2/PVP‐1–5, respectively. In addition, five pale yellow HCMP NW/PVP films containing 0.4, 1.2, 1.9, 2.7, and 3.9 wt.% HCMP NW were fabricated and denoted as HCMP NW/PVP‐1–5, respectively.

While the top view SEM images of MnO_2_ NW/PVP‐1–2, MnO_2_@CMP‐2/PVP‐1–2, and HCMP NW/PVP‐1–2 films exhibited flat surfaces, MnO_2_ NW, MnO_2_@CMP‐2, and HCMP materials were detected in the MnO_2_ NW/PVP‐3–5, MnO_2_@CMP‐2/PVP‐3–5, and HCMP NW/PVP‐3–5 films (Figure S7, Supporting Information). The thicknesses of MnO_2_ NW/PVP, MnO_2_@CMP‐2/PVP, and HCMP NW/PVP films were measured to be 25 µm (Figure S8, Supporting Information). The IR spectra of films showed exclusively the C─H, C═O, C─N, and C─O vibration peaks of the PVP matrix at 2891–2957, 1659, 1450, and 1283 cm^−1^, respectively,^[^ ^25^ ^]^ due to the relatively small amount of MnO_2_ NW, MnO_2_@CMP‐2, and HCMP in the films (Figure S9, Supporting Information). While the PXRD patterns of MnO_2_ NW/PVP‐1, MnO_2_@CMP‐2/PVP‐1, and HCMP NW/PVP‐1–5 films showed broad diffraction peaks at 2*θ* of 10.3° and 20.7°, corresponding to the PVP matrix,^[^ ^26^ ^]^ those of MnO_2_ NW/PVP‐2–5 and MnO_2_@CMP‐2/PVP‐2–5 films revealed the original diffraction peaks of α‐MnO_2_ (Figure S10, Supporting Information).

The triboelectric performance of MnO_2_ NW/PVP, MnO_2_@CMP‐2/PVP, and HCMP/PVP films with an area of 2 cm × 2 cm was studied (Figure 5b–e; Figure S11, Supporting Information). As a tribonegative material, perfluoroalkoxy alkanes (PFA) were used.^[^ ^27^ ^]^ In the case of pristine PVP and MnO_2_ NW/PVP films, as the amount of MnO_2_ NW increased, the output peak‐to‐peak voltages (V_p‐p_) gradually increased from 234 V (PVP) to 302 (MnO_2_ NW/PVP‐1), 329 (MnO_2_ NW/PVP‐2), and 365 V (MnO_2_ NW/PVP‐3), respectively (Figure 5b). The corresponding output currents (I_p‐p_) increased from 16.8 µA (PVP) to 21.1 (MnO_2_ NW/PVP‐1), 24.3 (MnO_2_ NW/PVP‐2), and 26.0 µA (MnO_2_ NW/PVP‐3), respectively (Figure S11, Supporting Information). Then, the V_p‐p_ of MnO_2_ NW/PVP‐4 and MnO_2_ NW/PVP‐5 films decreased to 331 and 265 V, respectively, with a decrease of I_p‐p_ to 24.1 and 19.6 µA. In the case of HCMP NW/PVP films, as the amount of HCMP NW increased, V_p‐p_ gradually increased from 339 V (HCMP NW/PVP‐1) to 385 (HCMP/PVP‐2) and 456 V (HCMP/PVP‐3) with an increase of I_p‐p_ from 23.8 to 25.9 and 31.7 µA (Figure 5c; Figure S11, Supporting Information). Then, the V_p‐p_ of HCMP NW/PVP‐4 and HCMP NW/PVP‐5 films decreased to 359 and 306 V, respectively, with a decrease of I_p‐p_ to 25.8 and 21.4 µA.

In comparison, as the amount of MnO_2_@CMP‐2 increased, V_p‐p_ significantly increased from 375 V (MnO_2_@CMP‐2/PVP‐1) to 507 (MnO_2_@CMP‐2/PVP‐2) and 576 V (MnO_2_@CMP‐2/PVP‐3) with an increase of I_p‐p_ from 25.9 to 36.1 and 39.6 µA (Figure 5d; Figure S11, Supporting Information). Then, the V_p‐p_ of MnO_2_@CMP‐2/PVP‐4 and MnO_2_@CMP‐2/PVP‐5 films decreased to 359 and 306 V, respectively, with a decrease of I_p‐p_ to 36.1 and 24.4 µA. These observations indicated that the MnO_2_ NW/PVP‐3, HCMP NW/PVP‐3, and MnO_2_@CMP‐2/PVP‐3 are the best films. Among these films, the triboelectric performance was gradually enhanced in the order of MnO_2_ NW/PVP‐3 < HCMP NW/PVP‐3 < MnO_2_@CMP‐2/PVP‐3 (Figure 5e).

**Figure 6:**
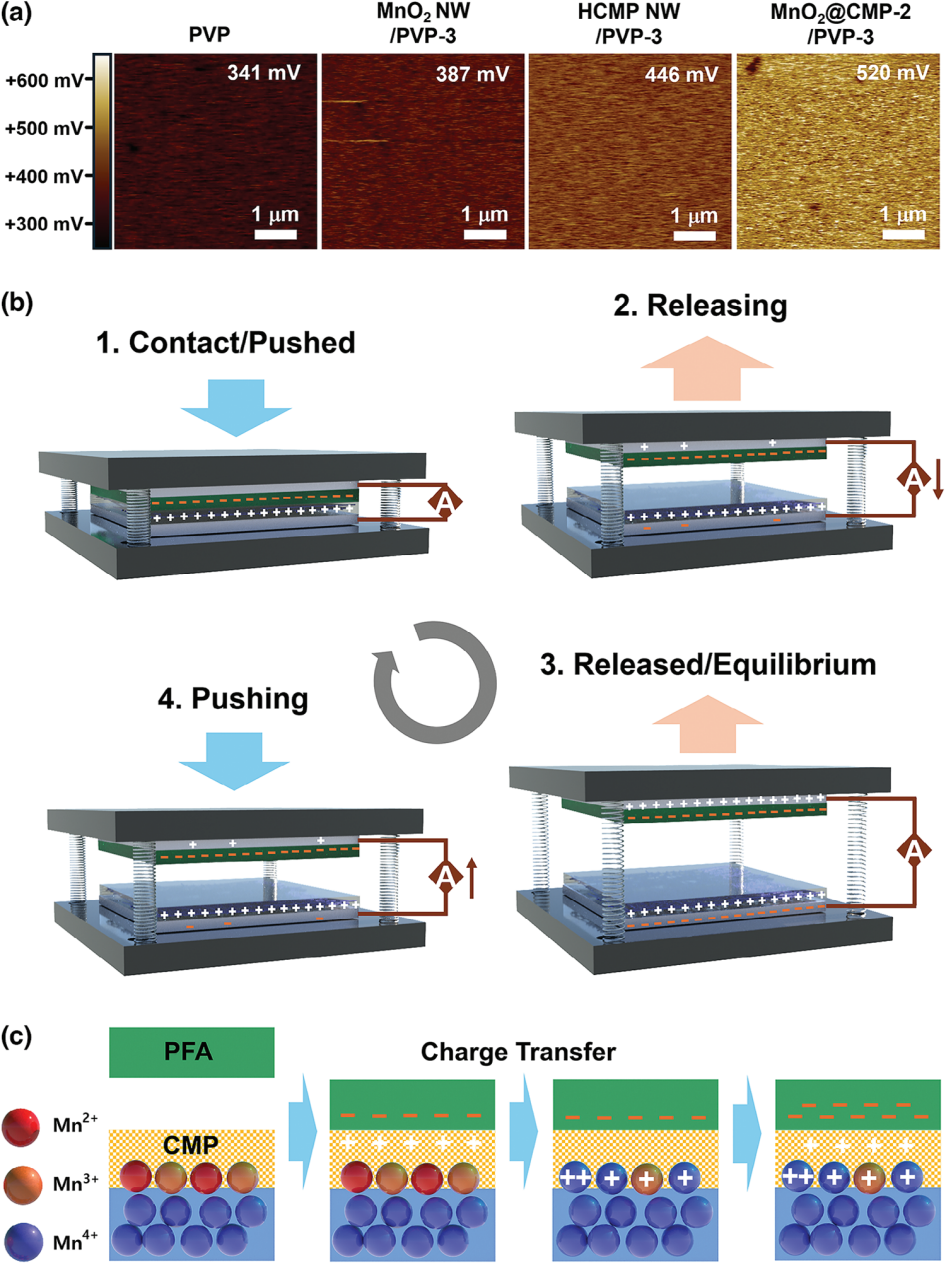
a) KPFM images of PVP, MnO_2_ NW/PVP‐3, HCMP NW/PVP‐3, and MnO_2_@CMP‐2/PVP‐3 films. b) A mechanism of the triboelectric energy harvesting process by a tribopositive a MnO_2_@CMP‐2/PVP film and a tribonegative PFA film. c) A suggested mechanism of the MnO_2_ NW‐assisted tribopositive performance of MnO_2_@CMP‐2.

According to Kelvin probe force microscopy (KPFM), the surface potentials gradually increased from 341 mV (PVP) to 387 (MnO_2_ NW/PVP‐3), 446 (HCMP NW/PVP‐3), and 520 mV (MnO_2_@CMP‐2/PVP‐3), matching with the observed trend of the tribopositive performance of films (**Figure** **6**a). The mechanism of electricity generation through triboelectrification has been reported in the literature (Figure 6b).^[^ ^28^ ^]^ At the contact of the pressed two films, electrons transfer from the tribopositive MnO_2_@CMP‐2/PVP‐3 film to the tribonegative PFA film. In the releasing state, electrons flow from the supporting metal electrode of a PFA film to the supporting metal electrode of an MnO_2_@CMP‐2/PVP‐3 film, reaching an equilibrium state. During the re‐pushing state of two films, electrons flow back from the supporting metal electrode of an MnO_2_@CMP‐2/PVP‐3 film to the supporting metal electrode of a PFA film. This process was repeated in the pushing/releasing cycles.

We propose the following mechanistic principle for the enhanced tribopositive performance of MnO_2_@CMP‐2 (Figure 6c). When the PFA film is in contact with MnO_2_@CMP‐2, electrons transfer from the tribopositive CMP to the PFA. The surface Mn^2+^ (indicated as red balls in Figure 6c) and Mn^3+^ (indicated as orange balls in Figure 6c) species can be converted to Mn^4+^ (indicated as blue balls in Figure 6c) through electron transfer to the CMP layers. With the assistance of the surface Mn^2+^ and Mn^3+^ species in MnO_2_ NW, the CMP layers can further enhance their role as tribopositive materials.

**Figure 7:**
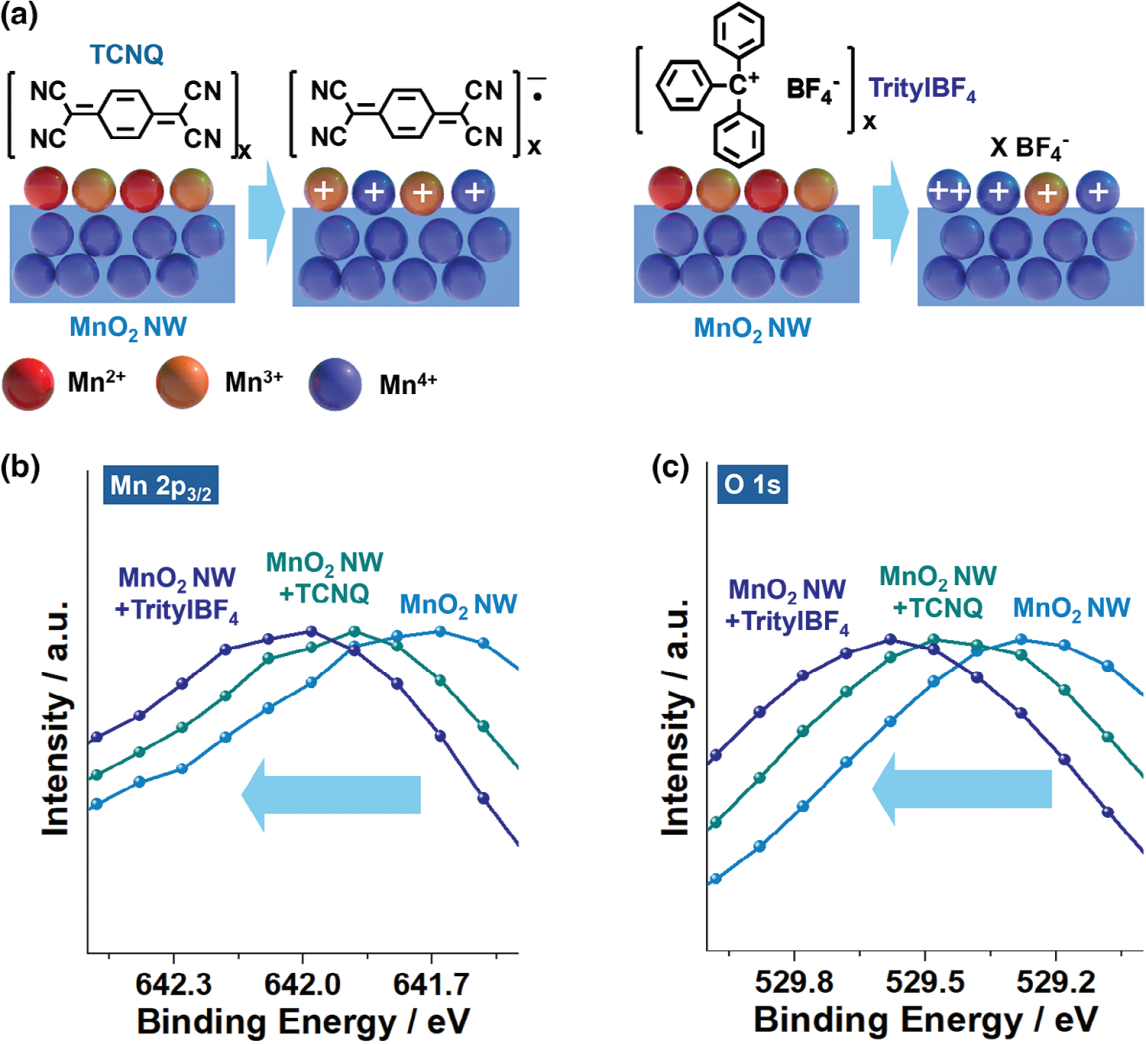
a) Model reactions of MnO_2_ NW with tetracyanoquinone (TCNQ) and trityl tetrafluoroborate (TritylBF_4_). XPS b) Mn 2p_3/2_ and c) O 1s orbital spectra of MnO_2_ NW with normalized intensities before and after treatment of MnO_2_ NW with TCNQ and TritylBF_4_ (Refer to Figures S12 and S13, Supporting Information for detailed analysis).

To investigate the possible redox behaviors of the surface Mn^2+^ and Mn^3+^ species of MnO_2_ NW, we conducted the following model studies (**Figure** **7**; Figures S12–S14, Supporting Information). Because PFA is a polymeric material and the generation of cationic charges on CMP through triboelectrification is an instantaneous event, XPS studies on the changes of the surface Mn^2+^ and Mn^3+^ species of MnO_2_ NW are technically limited. Thus, we treated MnO_2_ NW with tetracyanoquinone (TCNQ), as an electron‐deficient model compound,^[^ ^29^ ^]^ and trityl tetrafluoroborate (TritylBF_4_),^[^ ^30^ ^]^ as a model carbocation (Figure 7a).

When the MnO_2_ NW was treated with TCNQ and TritylBF_4_, the XPS Mn 2p orbital peaks significantly shifted to the higher energy region by 0.21 and 0.35 eV, respectively, indicating the conversion of the surface Mn^2+^ and Mn^3+^ species to Mn^3+^ and Mn^4+^ species. The detailed analysis indicated an increase in the Mn^4+^/(Mn^2+^ + Mn^3+^) values from 0.31 to 0.42 and 0.43 after the treatment of the MnO_2_ NW with TCNQ and TritylBF_4_, respectively (Figure 7b; Figure S12, Supporting Information). In addition, the O 1s orbital peaks of Mn─O─Mn species also shifted to the higher energy region by 0.18 and 0.32 eV, respectively, indicating the increased oxidation state of Mn species (Figure 7c; Figure S13, Supporting Information). Electron paramagnetic resonance (EPR) spectra confirmed the generation of anionic TCNQ radicals and trityl radicals (Figure S14, Supporting Information). These observations indicate that the surface Mn^2+^ and Mn^3+^ species of MnO_2_ NW can be converted to Mn^3+^ and Mn^4+^ species through the interaction with electron‐deficient materials and carbocation species.

**Figure 8:**
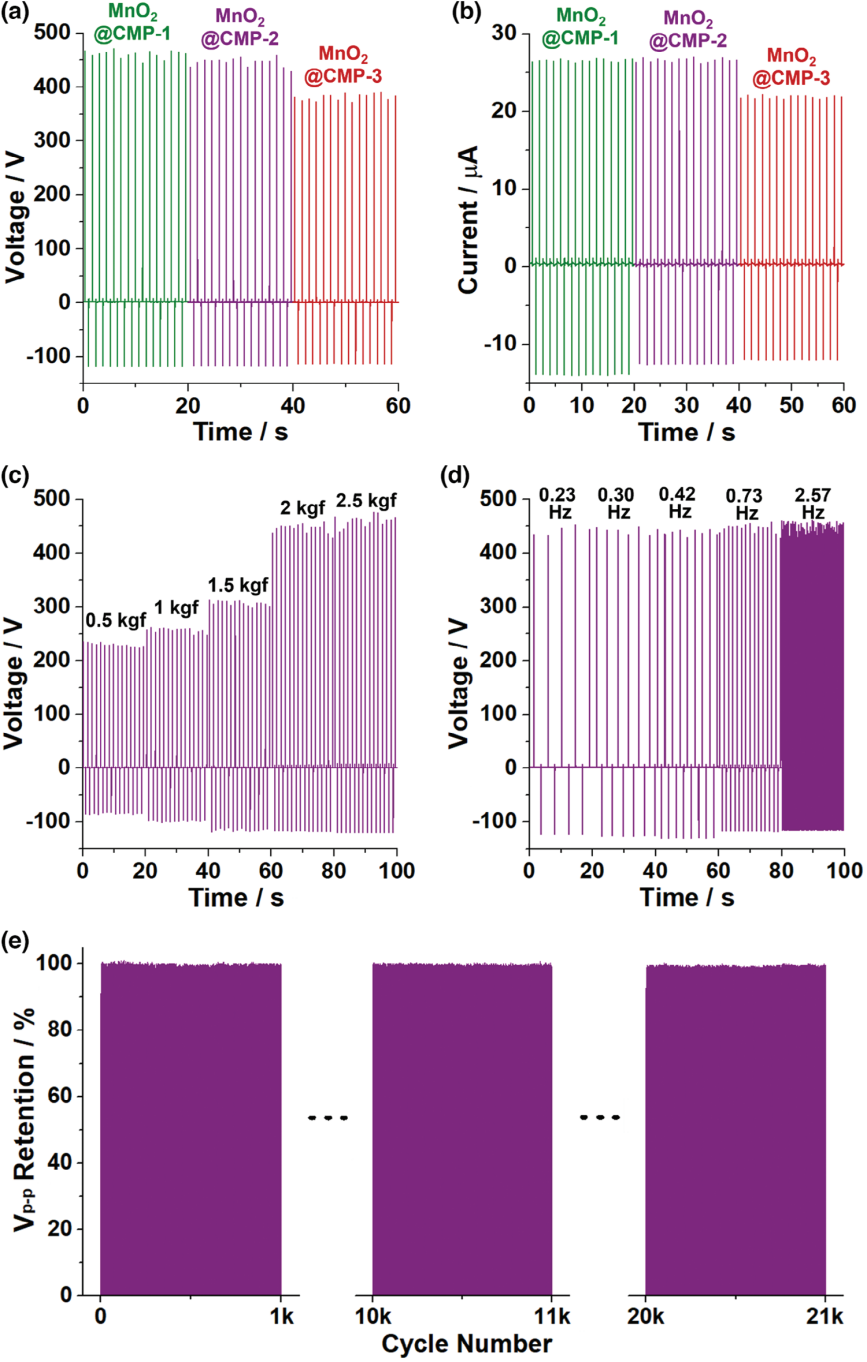
a,b) CMP layers thickness‐dependent output voltages and currents of MnO_2_@CMP/PVP films (Refer to Figure S15, Supporting Information for more studies). c) Pushing force and d) pushing frequency‐dependent output voltages and e) the retention of output voltages of MnO_2_@CMP‐2/PVP‐3 film (standard working conditions: a working area of 2 cm × 2 cm, a pushing force of 2 kgf, a pushing frequency of 0.73 Hz, RH 50%). Refer to Figures S16 and S17, Supporting Information for the output currents of corresponding films.

The thickness effect of CMP layers on the triboelectric performance of MnO_2_@CMPs was investigated (**Figure** **8**a,b; Figure S15, Supporting Information). The MnO_2_@CMP‐1 with 3.5 ± 0.4 nm thick CMP layers showed slightly better triboelectric performance with V_p‐p_ of 590 V and I_p‐p_ of 40.8 µA than the MnO_2_@CMP‐2 with 6.9 ± 0.7 nm thick CMP layers displaying V_p‐p_ of 576 V and I_p‐p_ of 39.6 µA. In comparison, the MnO_2_@CMP‐3 with 11.4 ± 0.7 nm thick CMP layers showed significantly lower V_p‐p_ of 505 V and I_p‐p_ of 34.2 µA. These results indicate that the thickness of CMP layers should be sufficiently thin at a sub‐10 nm scale for efficient charge transfer to the inner MnO_2_ NW during the triboelectrification process.

The working condition‐dependent triboelectric performance of the MnO_2_@CMP‐2/PVP‐3 was investigated (Figure 8c,d; Figures S16 and S17, Supporting Information). At relative humidities (RH) of 30% and 50%, the MnO_2_@CMP‐2/PVP‐3 exhibited similar triboelectric performance with V_p‐p_ of 554 and 576 V, respectively, and I_p‐p_ of 39.2 and 39.6 µA, respectively (Figure S16, Supporting Information). When RH increased to 80%, the V_p‐p_ and I_p‐p_ significantly dropped to 310 V and 28.7 µA, respectively.

The triboelectric performance of MnO_2_@CMP‐2/PVP‐3 was sensitive to the pushing force (Figure 8c; Figure S17, Supporting Information). As the pushing forces increased from 0.5 to 1, 1.5, 2, and 2.5 kgf, the V_p‐p_ increased from 322 to 363, 431, 576, and 600 V, respectively, with an increase of I_p‐p_ from 22.5 to 24.6, 30.3, 39.6, and 42.0 µA. In contrast, the MnO_2_@CMP‐2/PVP‐3 exhibited similar triboelectric performance across a pushing frequency range of 0.23–2.57 Hz, maintaining V_p‐p_ of 575–576 V and I_p‐p_ of 39.6–39.8 µA (Figure 8d; Figure S17, Supporting Information).

The durability of MnO_2_@CMP‐2/PVP‐3 was studied through cycling tests (Figure 8e). At the optimized working conditions (a pushing force of 2 kgf, a pushing frequency of 0.73 Hz, RH 50%), the MnO_2_@CMP‐2/PVP‐3 maintained the original triboelectric performance in the range of 98.8–100% over 21 000 cycling tests.

**Figure 9:**
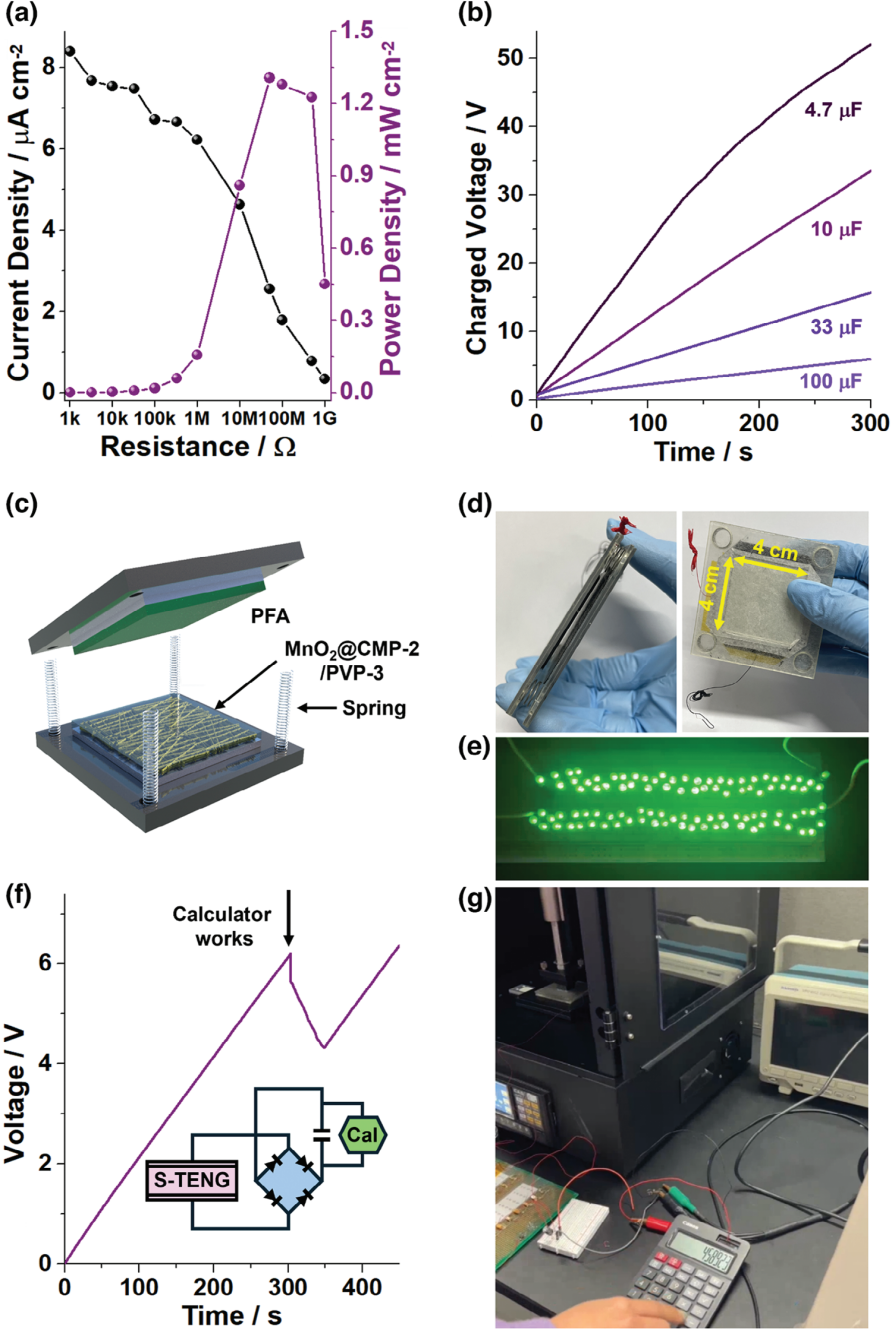
a) Resistance‐dependent current and power densities (a film area of 2 cm × 2 cm, a pushing force of 2 kgf) and b) charged voltages of electrolytic capacitors by the triboelectric performance of a MnO_2_@CMP‐2/PVP‐3 film. c) An illustration and d) a photograph of an S‐TENG fabricated using MnO_2_@CMP‐2/PVP‐3 and PFA films. e,f,g) Demonstration of S‐TENGs as power sources to turn on 100 green LED bulbs and to operate an electronic calculator (working conditions: a film area of 4 cm × 4 cm, a pushing force of 2 kgf, a pushing frequency of 0.73 Hz, RH 50%).

Resistance‐dependent current and power densities generated by triboelectric MnO_2_@CMP‐2/PVP‐3 films were measured, showing a maximum power density (P_max_) of 1.31 mW cm^−2^ at a resistance of 5 × 10^7^ Ω (**Figure** **9**a). Very recently, microporous organic materials such as covalent organic frameworks (COFs) and CMPs have been applied as triboelectric materials, showing V_p‐p_ of 40–815 V and P_max_ of 0.364 µW cm^−2^–0.824 mW cm^−2^ (Table S1, Supporting Information).^[^ ^8^, ^11^ ^]^ In addition, metal–organic framework (MOF)‐based triboelectric materials showed the V_p‐p_ of 62–658 V and P_max_ of 0.968 µW cm^−2^–0.508 mW cm^−2^ (Table S2, Supporting Information).^[^ ^9^ ^]^ In this regard, the triboelectric performance of the MnO_2_@CMP‐2/PVP‐3 film, showing V_p‐p_ of 576 V and P_max_ up to 1.31 mW cm^−2^, is quite promising.

The triboelectric MnO_2_@CMP‐2/PVP‐3 film could be utilized to charge electrolytic capacitors (Figure 9b). After 5 min, voltages of 50, 32, 15, and 5 V were achieved for 4.7, 10, 33, and 100 µF capacitors, respectively. Spring‐assisted triboelectric nanogenerators (S‐TENGs) were fabricated using MnO_2_@CMP‐2/PVP‐3 and PFA films (Figure 9c,d). It was confirmed that the S‐TENG could work as a power supply to illuminate 100 green LED bulbs (Figure 9e; Movie S2, Supporting Information). Moreover, after a battery was removed from an electronic calculator, the S‐TENG was connected as a power supply. A capacitor charged to 6 V for 300 s using the S‐TENG could be used to operate a calculator (Figure 9f,g; Movie S3, Supporting Information).

## 3 Conclusion

In conclusion, this work shows that the triboelectric performance of CMP materials can be significantly enhanced with the assistance of MnO_2_ NW. As a cooperative mechanistic principle for the triboelectrification of MnO_2_@CMP, the possible redox role of the surface Mn^2+^ and Mn^3+^ species of MnO_2_ NW was suggested. Especially, the CMP shell thickness of MnO_2_@CMP was also critical and should be a sub‐10 nm scale to ensure efficient tribopositive materials. Model studies supported the possible electron transfer from the surface Mn^2+^ and Mn^3+^ species of MnO_2_ NW to organic materials. Based on the observed results of this work, we believe that various redox‐active inorganic material@CMP systems can be developed as effective triboelectric materials.

## Conflict of Interest

The authors declare no conflict of interest.

## Supporting information



Supporting Information

Supplemental Movie 1

Supplemental Movie 2

Supplemental Movie 3

## Data Availability

The data that support the findings of this study are available in the supplementary material of this article.
