# Supplementary material for: MnO2 Nanowires with Sub‐10 nm Thick Conjugated Microporous Polymers as Synergistic Triboelectric Materials
Source: Adv Sci (Weinh). 2024 Nov 3;11(48):2409917. doi: 10.1002/advs.202409917 (PMC11672312; doi:10.1002/advs.202409917)
Supplement: Supplementary file 1 — Supporting Information [file ADVS-11-2409917-s002.docx]

Copyright WILEY-VCH Verlag GmbH & Co. KGaA, 69469 Weinheim, Germany, 2024.

Supporting Information

MnO_2_ Nanowires with Sub-10 nm Thick Conjugated Microporous Polymers as Synergistic Triboelectric Materials

*Hanbyeol Jung, Dong-Min Lee, Jina Park, Taeho Kim, Sang-Woo Kim,* and Seung Uk Son**

**Experimental Section**

**General Information:** SEM images were obtained by a JSM-7100F instrument at the Chiral Material Core Facility Center of Sungkyunkwan University. TEM and EDS-elemental mapping images were obtained by a JEM-2100F intrument. HR-TEM images were obtained by a JEM-ARM200F instrument. PXRD patterns were obtained by a Rigaku MAX-2200 instrument. N_2_ adsorption-desorption isotherm curves were obtained at 77 K by a Micromeritics ASAP2020 equipment. The pore size distribution diagrams were obtained through the analysis of N_2_ adsorption-desorption isotherm curves based on the NL-DFT method. IR spectra were obtained by a Bruker VERTEX 70 FT-IR spectrometer at the Chiral Material Core Facility Center of Sungkyunkwan University. Solid sate ^13^C NMR spectrum was obtained by a 500 MHz Bruker ADVANCE II NMR spectrometer. XPS spectra were obtained by a Thermo VG spectrometer. TGA curves were obtained by a TG/DTA-7300 instrument. EPR spectra were obtained by a EMXplus-9.5/12/P/L spectrometer with a microwave frequency of 9.84 GHz at 298 K.

**Preparation of MnO_2_ NW, MnO_2_@CMPs, and HCMP NW**

MnO_2_ nanowires were prepared by the synthetic method reported in the literature.^[1]^ In this work, the following procedures were applied. After acetic acid (1.3 mL, 23 mmol) and potassium acetate (1.3 g, 13 mmol) dissolved in distilled water (10 mL) were added to a 100 mL two neck Schlenk flask, the solution was cooled using an ice bath. After aqueous H_2_O_2_ solution (34.5 wt%, 0.2 mL) was diluted through mixing with distilled water (10 mL), it was added dropwise to the aqueous acetic acid/potassium acetate solution. After KMnO_4_ (1.6 g, 10 mmol) was dissolved in distilled water (37.5 mL), the solution was added to the reaction mixture. After strring at 110 ^o^C for 24 h, the reaction mixture was cooled to room temperature. The dark brown MnO_2_ NW was retrieved by filtration, washed with water (300 mL) and ethanol (300 mL), and dried uder vacuum at 80 ^o^C for 6 h.

For the preparation of MnO_2_@CMP-2, MnO_2_ NW (0.20 g), triethylamine (40 mL), CuI (3.9 mg, 20 μmol), and (PPh_3_)_2_PdCl_2_ (14 mg, 20 μmol) were added to a flame-dried 100 mL Schlenk flask. The reaction mixture was sonciated at room temperature for 30 min. After 1,3,5-triethynylbenzene (30 mg, 0.20 mmol) and 1,4-diiodobenzene (99 mg, 0.30 mmol) were dissolved in distilled toluene (20 mL), the solution was added to the reaction mixture. After being stirred at 80 ^o^C for 18 h, the reaction mixture was cooled to room temperature. The MnO_2_@CMP-2 was retrieved through centrifugation, washed with a mixture of ethanol (20 mL), methanol (20 mL), and dichloromethane (10 mL) six times, and dried under vacuum.

For the preparation of MnO_2_@CMP-1, the same synthetic procedures of MnO_2_@CMP-2 were applied except using CuI (2.0 mg, 10 μmol), (PPh_3_)_2_PdCl_2_ (7.1 mg, 10 μmol), 1,3,5-triethynylbenzene (15 mg, 0.10 mmol), and 1,4-diiodobenzene (50 mg, 0.15 mmol). For the preparation of MnO_2_@CMP-3, the same synthetic procedures of MnO_2_@CMP-2 were applied except using CuI (7.7 mg, 40 μmol), (PPh_3_)_2_PdCl_2_ (28 mg, 40 μmol), 1,3,5-triethynylbenzene (60 mg, 0.40 mmol), and 1,4-diiodobenzene (0.20 g, 0.60 mmol).

For the preparation of HCMP NW, MnO_2_@CMP-2 (0.10 g) was added to a mixture of aqueous HCl (35.0~37.0%, 7.5 mL), distilled water (5 mL), and methanol (35 mL) in a 50 mL Falcon Tube. After the dispersion was stirred for 8 h at room temperature, the solid was retrieved by centrifugation, washed with a mixture of methanol (35 mL) and distilled water (15 mL) five times, and dried under vacuum.

**Model studies: the treatment of MnO_2_ NW with TCNQ and TritylBF_4_.**

After 7,7,8,8-tetracyanoquinodimethane (TCNQ, 10 mg, 49 μmol) was dissolved in a distilled THF (12 mL) in a 20 mL vial, MnO_2_ NW (43.5 mg) was added. The suspension was stirred at room temperature for 12 h. After the solvent was evaporated, the solid was dribed under vacuum and analyzed by XPS.

Tritylium tetrafluoroborate (TritylBF_4_, 16 mg, 49 μmol) was dissolved in a distilled methylene chloride (12 mL) in a 20 mL vial. After MnO_2_ NW (43.5 mg) was added, the suspension was stirred at room temperature for 12 h. After the solvent was evaporated, the solid was dried under vacuum and analyzed by XPS.

**Preparation of PVP, MnO_2_ NW/PVP, MnO_2_@CMP/PVP, and HCMP NW/PVP films**

For the preparation of MnO_2_ NW/PVP-1~5 films, polyvinylpyrrolidone (PVP, Mw: 1,300,000, Aldrich. Co., 10 g) was dissolved in ethanol (40 g) by stirring at 50 ^o^C for 4 h in a 70 mL vial to form 20 wt% PVP solution. After 20 wt% PVP solution (4 mL) was taken from the mother solution, it was transferred to a 20 mL vial that was pre-weighed. After ethanol was evaporated, the PVP powder in a 20 mL vial was dried at 80 ^o^C under vacuum for 6 h. Through this process, the amount of PVP powder in the 4 mL of PVP solution was determined to be 0.724 g.

For the preparation of PVP films, a polyvinylchloride mat (PVC, Hyundai Co., 20 cm × 20 cm) was loaded on a Knife Coating Device (model: KP-3000 V, Kipae E&T Co., Ltd). After 20 wt% PVP solution (4 mL) was loaded on a PVC mat, the film with the thickness of 30 μm was fabricated using a micrometer film applicator (model: 1117/200, Jongro Industrial Co., Ltd) and a doctor blade. After drying at room temeprature for 24 h, the PVP film was detached using tweezers and cut in to pieces with the areas of 2 cm × 2 cm or 4 cm × 4 cm.

For the preparation of MnO_2_ NW/PVP-1 films having 1 wt% MnO_2_ in PVP, MnO_2_ NW (7.3 mg), ethanol (1 mL), and 20 wt% PVP solution (4 mL) were added to an agate mortar. After the mixture was ground, it was loaded on a PVC mat. The film with the thickness of 30 μm was fabricated using a micrometer film applicator (model: 1117/200, Jongro Industrial Co., Ltd) and a doctor blade. After drying at room temeprature for 24 h, the MnO_2_ NW/PVP-1 film was detached using tweezers and cut in to pieces with the areas of 2 cm × 2 cm or 4 cm × 4 cm. For the preparation of MnO_2_ NW/PVP-2 (3 wt% MnO_2_ NW), MnO_2_ NW/PVP-3 (5 wt% MnO_2_ NW), MnO_2_ NW/PVP-4 (7 wt% MnO_2_ NW), and MnO_2_ NW/PVP-5 (10 wt% MnO_2_ NW), the same fabrication procedures of MnO_2_ NW/PVP-1 films were applied except using 22.4, 38.1, 54.5, and 80.4 mg MnO_2_ NW, respectively.

For the preparation of MnO_2_@CMP-2/PVP-1, MnO_2_@CMP-2/PVP-2, MnO_2_@CMP-2/PVP-3, MnO_2_@CMP-2/PVP-4, and MnO_2_@CMP-2/PVP-5 films, the same fabrication procedures of MnO_2_ NW/PVP-1 films were applied except using 10.2, 31.5, 54.0, 78.0, and 116.8 mg MnO_2_@CMP-2, respectively. The contents of MnO_2_ in the MnO_2_@CMP-2/PVP-1, MnO_2_@CMP-2/PVP-2, MnO_2_@CMP-2/PVP-3, MnO_2_@CMP-2/PVP-4, and MnO_2_@CMP-2/PVP-5 films were calculated to be 1, 3, 5, 7, and 10 wt%, respectively. The contents of CMP in the MnO_2_@CMP-2/PVP-1, MnO_2_@CMP-2/PVP-2, MnO_2_@CMP-2/PVP-3, MnO_2_@CMP-2/PVP-4, and MnO_2_@CMP-2/PVP-5 films were calculated to be 0.39, 1.17, 1.94, 2.72, and 3.89 wt%, respectively.

For the preparation of HCMP NW/PVP-1, HCMP NW/PVP-2, HCMP NW/PVP-3, HCMP NW/PVP-4, and HCMP NW/PVP-5 films, the same fabrication procedures of MnO_2_ NW/PVP-1 films were applied except using 2.8, 8.6, 14.4, 20.3, and 29.3 mg HCMP NW, respectively. The contents of HCMP NW in the HCMP NW/PVP-1, HCMP NW/PVP-2, HCMP NW/PVP-3, HCMP NW/PVP-4, and HCMP NW/PVP-5 films were calculated to be 0.39, 1.17, 1.94, 2.72, and 3.89 wt%, respectively.

For the preparation of MnO_2_@CMP-1/PVP-1~5 films, the same fabrication procedures of MnO_2_@CMP-2/PVP-1~5 films, respectively, were applied except using MnO_2_@CMP-1. For the preparation of MnO_2_@CMP-3/PVP-1~5 films, the same fabrication procedures of MnO_2_@CMP-2/PVP-1~5 films, respectively, were applied except using MnO_2_@CMP-3.

**Triboelectric performance of materials**

As tribopositive parts, PVP, MnO_2_ NW/PVP, MnO_2_@CMP/PVP, and HCMP NW/PVP films were cut into pieces with an area of 2 cm × 2 cm. The films were attached on the Au electrode (0.03 μm, 2 cm × 2 cm) of the printed circuit board (PCB, 3 cm × 3 cm, FR-4 Normal glass epoxy, thickness of 1.0T) using a carbon tape (2 cm × 2 cm). As a tribonegative part, Cu electrode (3M^TM^, conductive cooper foil tape, 3313 Copper, 1-1/2 in × 18 yd 3.0 mil, 2 cm × 2 cm) was attached to a polycarbonate support (2 cm × 2 cm). A perfluoroalkoxy alkanes (PFA, Alphafion, thickness of 25 μm, 2 cm × 2 cm) film^[2]^ was attached to the Cu electrode using a carbon tape (2 cm × 2 cm).

The Cu electrode of the tribonegative part was connected to the P6139B voltage probe (Tektronix, 500 MHz, 10 MΩ). Output currents were measured through the conversion using a low noise amplifier (DLPCA-299, FEMTO). Tribopositive and tribonegative materials were contacted using a pushing tester (JIPT-120, Junil Tech Co.). Relative humidity (RH) was scanned in the range of 30~80%. The pushing forces of 0.5, 1, 1.5, 2, and 2.5 kgf and the pushing frequencies of 0.23, 0.30, 0.42, 0.73, and 2.57 Hz were applied. For the cycling tests, a pushing force of 2 kgf and a pushing frequency of 0.73 Hz were applied at RH 50%.

**Measurement of surface potentials and power densities of films**

The surface potentials of PVP, MnO_2_ NW/PVP-3, HCMP NW/PVP-3, and MnO_2_@CMP-2/PVP-3 films were measured at a KPFM mode using an atomic force microscopy equipment (AFM, XE-100, Park Systems). A Cr/Pt-coated AFM probe (Multi75E-G, BudgetSensors) was used to scan the films at a noncontact mode. An amplitude of 2.0 V, a resonance frequency of 17 kHz, and a force constant of 3 N/m were aspplied.

The loading resistance-dependent power densities of a MnO_2_@CMP-2/PVP-3 film (an area of 2 cm × 2 cm) were measured as follows. Resistors with systematic resistances of 1 kΩ to 1 GΩ (Jeil Electronics) were connected to the triboelectric devices fabricated with MnO_2_@CMP-2/PVP-3 films. The output currents were measured using a current amplifier (FEMTO, DLPCA-200) and an oscilloscope (Tektronix, DPO3052). A pushing force of 2 kgf was applied with a pushing tester (SnM Co.). The powder denisties were calculated from the corresponding resistances and ouput currents

**Demonstration of S-TENGs as power suppliers to operate electronic devices**

A spring-assisted TENGs (S-TENGs) were fabricated as follows. The acrylic plates with an area of 7 cm × 7 cm were used as external supports. The internal acrylic structures with an area of 4 cm × 4 cm were loaded on the external supports. Al tapes with an area of 4 cm × 4 cm were attached on the internal acrylic structures as electrodes. Using carbon tapes with an area of 4 cm × 4 cm, a MnO_2_@CMP-2/PVP-3 film with an area of 4 cm × 4 cm) and a PFA film with an area of 4 cm × 4 cm were attached on the Al electrodes. Four springs were installed at the corners of the a MnO_2_@CMP-2/PVP-3 film/Al/an acrylic plate and the a PFA film/Al/an acrytlic plate.

In the charging tests of capactitors, the outputs of triboelectric devices were connected to a full wave bridge rectifier (Rectron Semiconductor Co, W04M) to convert AC outputs to DC ones. The rectified outputs were connected to Al electrolytic capacitors (Samyoung Electronics, SHL Series). A pushing force of 2 kgf was applied with a pushing tester (SnM Co.). The charged potentials of capacitors were measured using an electrometer (Keithley 6514).

In the lighting tests of LEDs, a pushing force of 2 kgf was applied with a pushing tester (SnM Co.) to a S-TENG. The outputs were rectified using a full wave bridge rectifier (Rectron Semiconductor Co., W04M) to obtain DC outputs. The recfified outputs were connected to 100 green LEDs (Photron Co., PV525-5A5D-NNISLA-Z).

In the operating tests of an electronic calculator, a S-TENG was operated using a pushing tester (SnM Co.) with a pushing force of 2 kgf. The outputs of the S-TENG were connected to a full wave bridge rectifier (Rectron Semiconductor Co., W04M) to obtain the rectified DC outputs. A 100 μF capacitor (Samyoung Electronics, SHL series) was charged through the connection to the recified DC outputs. After the battery of an electronic calculator (Cannon Co., AS-120II) was removed, the charged capacitor was connected to the calculator.

**Reference**

[1] a) J. C. Villegas, L. J. Garces, S. Gomez, J. P. Durand, S. L. Suib, *Chem. Mater.*, **2005**, *17*, 1910-1918. b) A. S. Poyraz, J. Laughlin, Z. Zec, *Electrochim. Acta*, **2019**, *305*, 423-432.

[2] J. Kim, H. Ryu, S. M. Kim, H. Y. Lee, A. Karami, D. Galayko, D. Kang, S. S. Kwak, H. -J. Yoon, P. Basset, S. -W. Kim, *Adv. Mater. Technol.*, **2024**, *9*, 2301309.

**Figure S1.** Photographs showing the generation of static electricity of MnO_2_@CMP-2 powder in a Falcon tube (refer to Movie S1 in the SI).


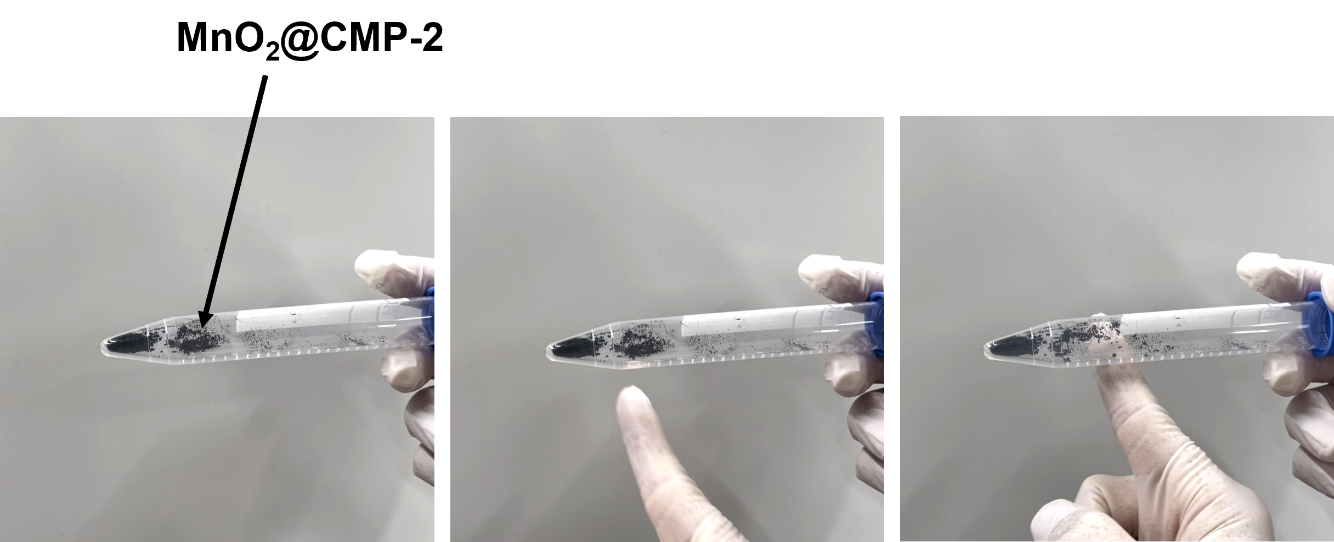


**Figure S2.** Thickness distribution diagrams of CMP layers in the (a) MnO_2_@CMP-1, (b) MnO_2_@CMP-2, and (c) MnO_2_@CMP-3.

**(a)**

**(c)**

**(b)**

**
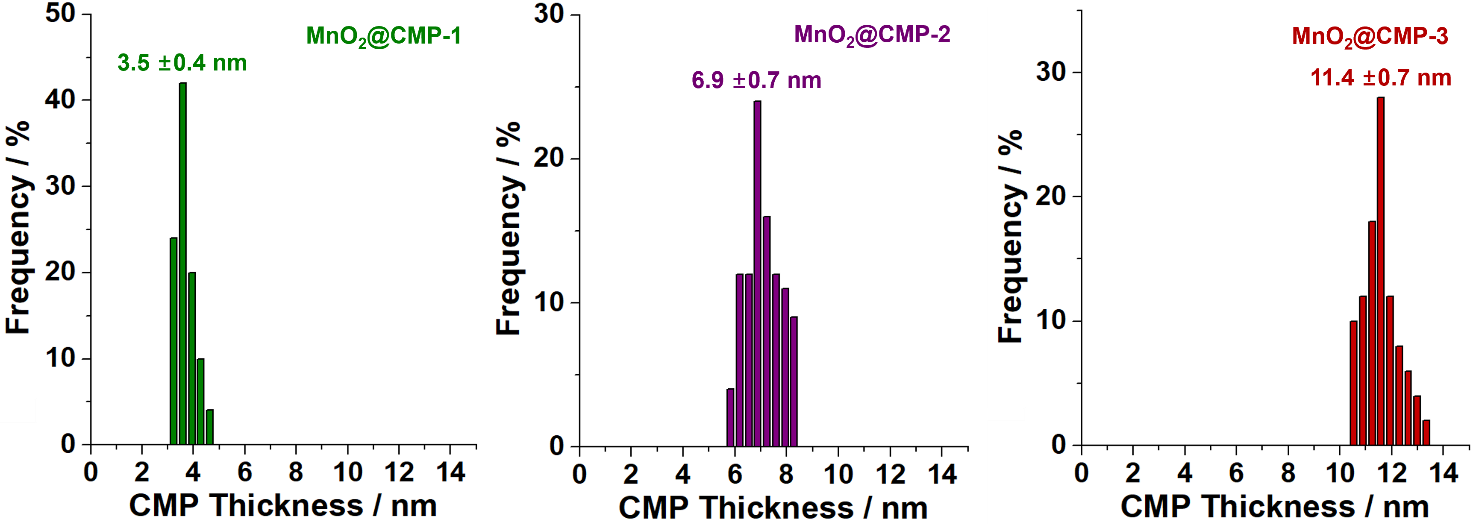
**

**Figure S3.** (a, c) Unnormalized and (b, d) normalized XPS Mn 2p orbital and O 1s orbital peaks of MnO_2_ NW, MnO_2_@CMP-1~3, and HCMP NW.

**
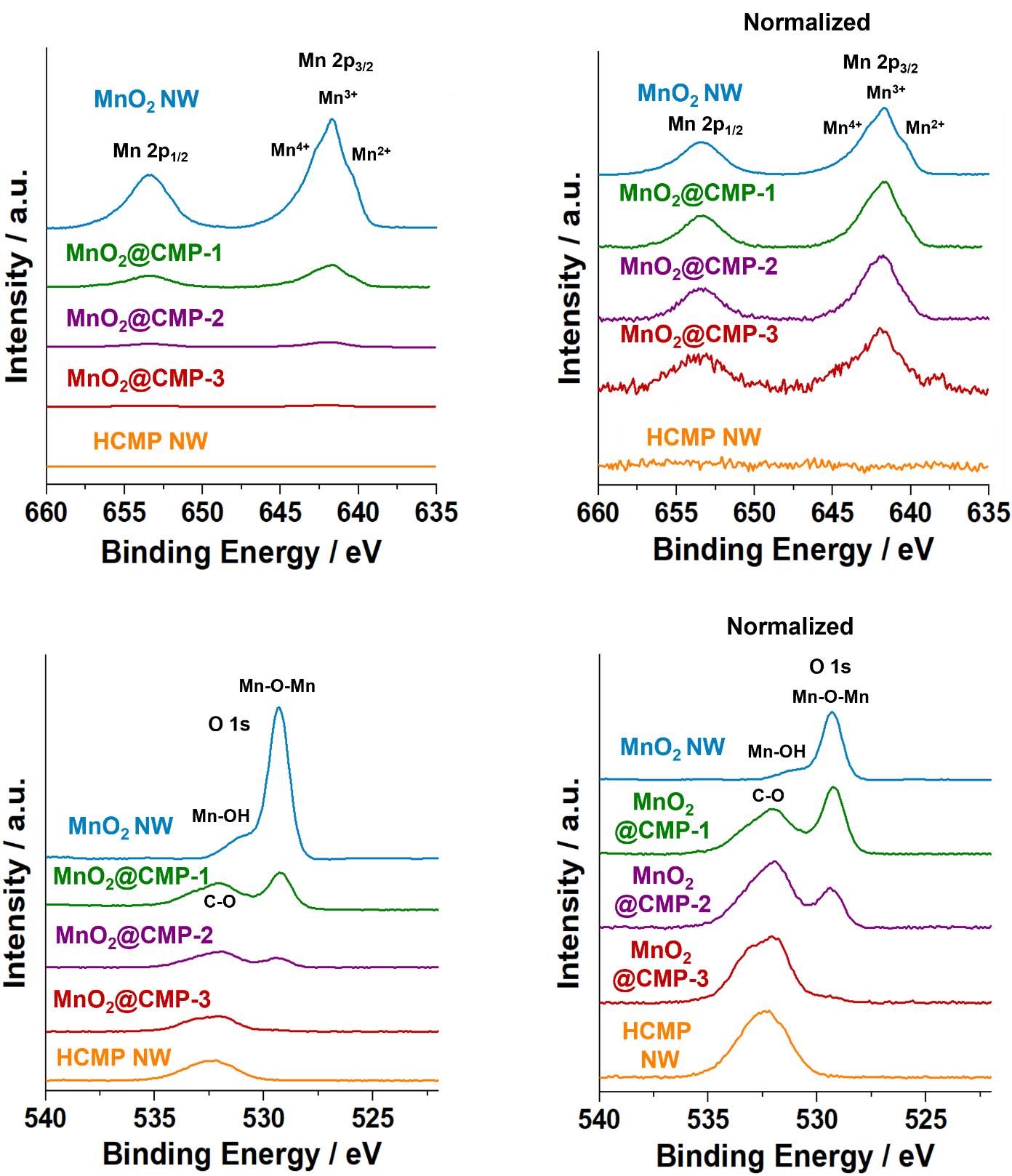
**

**(d)**

**(c)**

**(b)**

**(a)**

**Figure S4.** Analysis of XPS Mn 2p orbital peaks of MnO_2_ NW, MnO_2_@CMP-1~3, and HCMP NW.

**
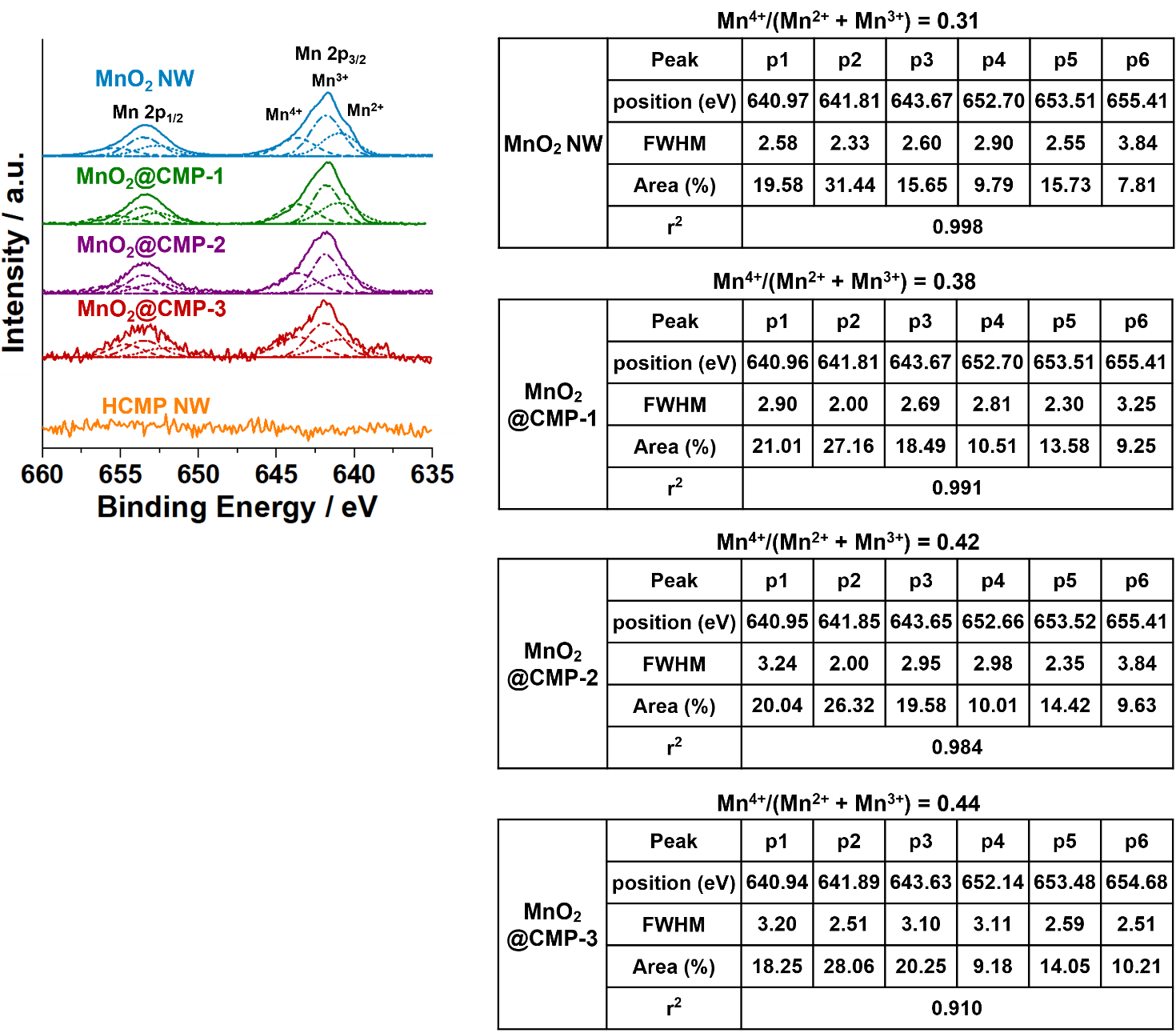
**

**Figure S5.** Analysis of XPS O 1s orbital peaks of MnO_2_ NW, MnO_2_@CMP-1~3, and HCMP NW.

**
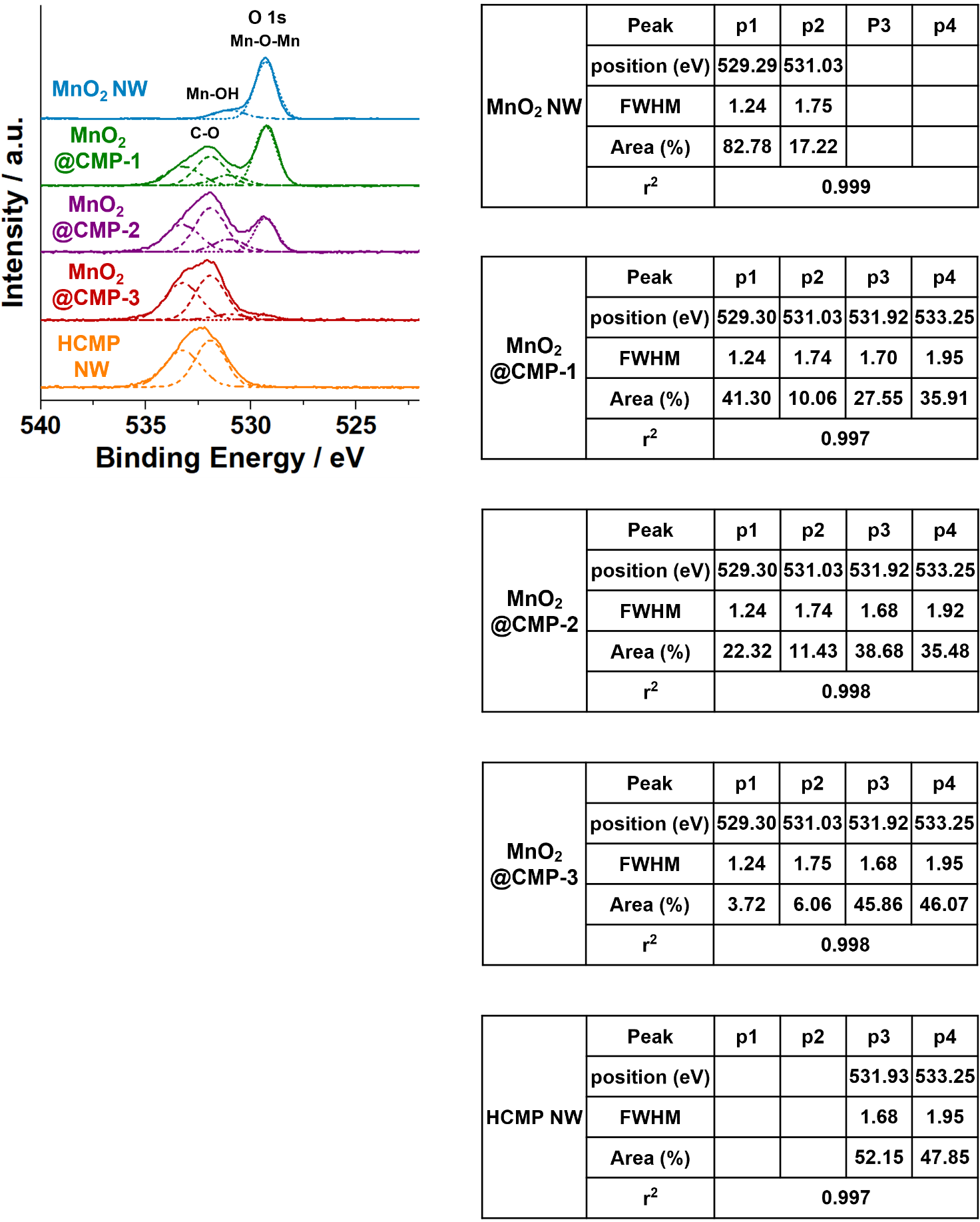
**

**Figure S6.** TGA curves of MnO_2_@CMP-1~3 under air.

**
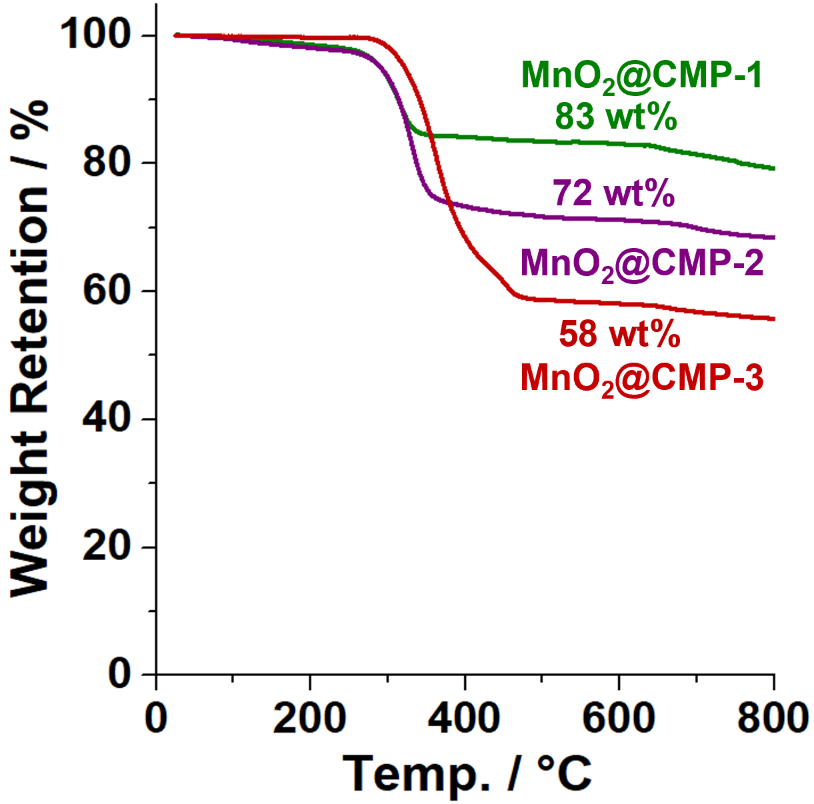
**

**Figure S7.** Top view SEM images of (a) MnO_2_ NW/PVP-1, (b) MnO_2_ NW/PVP-2, (c) MnO_2_ NW/PVP-3, (d) MnO_2_ NW/PVP-4, (e) MnO_2_ NW/PVP-5, (f) HCMP NW/PVP-1, (g) HCMP NW/PVP-2, (h) HCMP NW/PVP-3, (i) HCMP NW/PVP-4, (j) HCMP NW/PVP-5, (k) MnO_2_@CMP-1/PVP-1, (l) MnO_2_@CMP-1/PVP-2, (m) MnO_2_@CMP-1/PVP-3, (n) MnO_2_@CMP-1/PVP-4, (o) MnO_2_@CMP-1/PVP-5, (p) MnO_2_@CMP-2/PVP-1, (q) MnO_2_@CMP-2/PVP-2, (r) MnO_2_@CMP-2/PVP-3, (s) MnO_2_@CMP-2/PVP-4, (t) MnO_2_@CMP-2/PVP-5, (u) MnO_2_@CMP-3/PVP-1, (v) MnO_2_@CMP-3/PVP-2, (w) MnO_2_@CMP-3/PVP-3, (x) MnO_2_@CMP-3/PVP-4, and (y) MnO_2_@CMP-3/PVP-5 films.

**(e)**

**(d)**

**(c)**

**(b)**

**(a)**

**
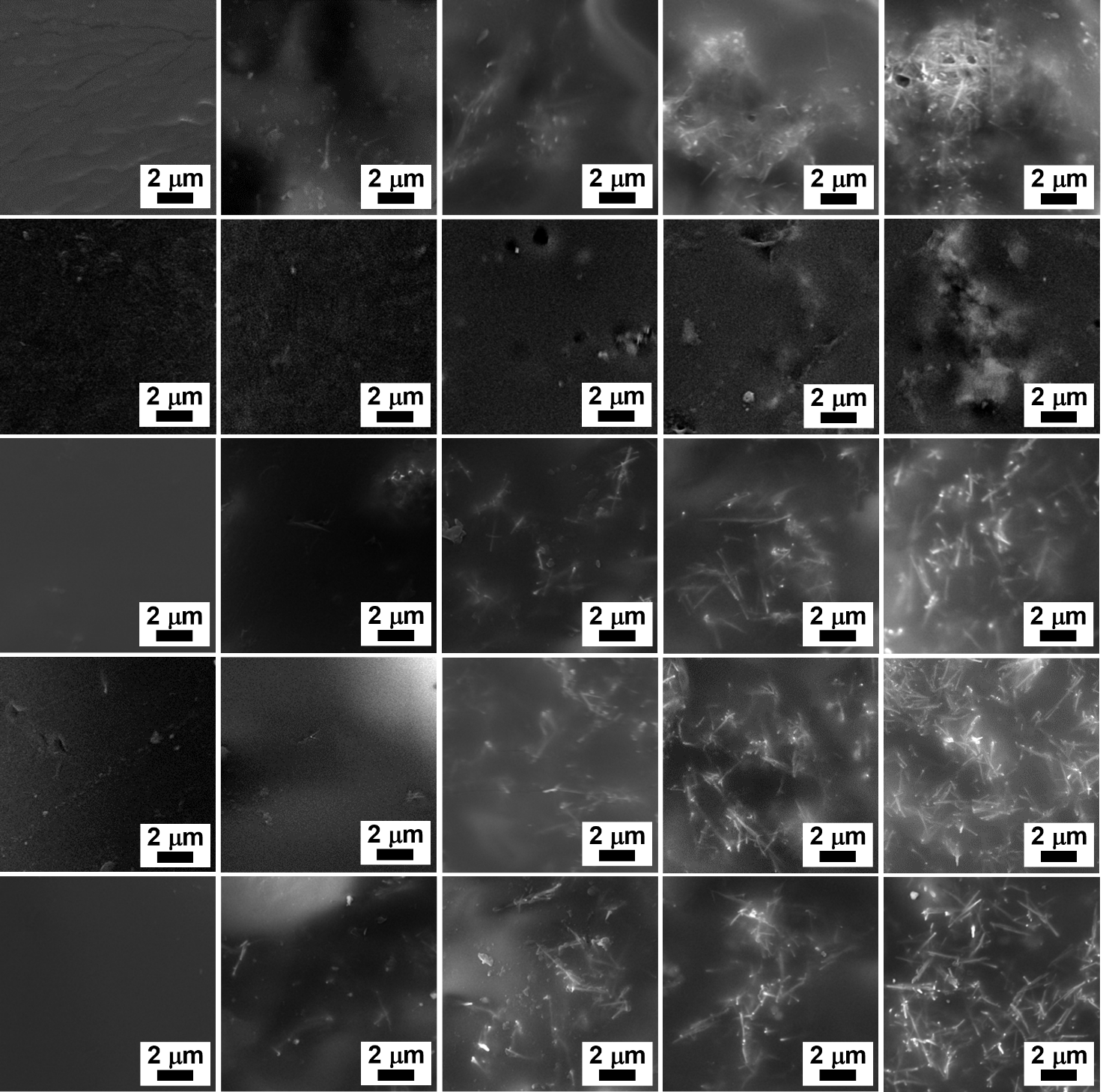
**

**(w)**

**(v)**

**(u)**

**(y)**

**(x)**

**(p)**

**(t)**

**(s)**

**(r)**

**(q)**

**(l)**

**(k)**

**(o)**

**(n)**

**(m)**

**(i)**

**(h)**

**(g)**

**(f)**

**(j)**

**Figure S8.** Side view SEM images of (a) PVP, (b) MnO_2_ NW/PVP-3, (c) HCMP NW/PVP-3, and (d) MnO_2_@CMP-2/PVP-3 films.

**(d)**

**(c)**

**(b)**

**(a)**

**
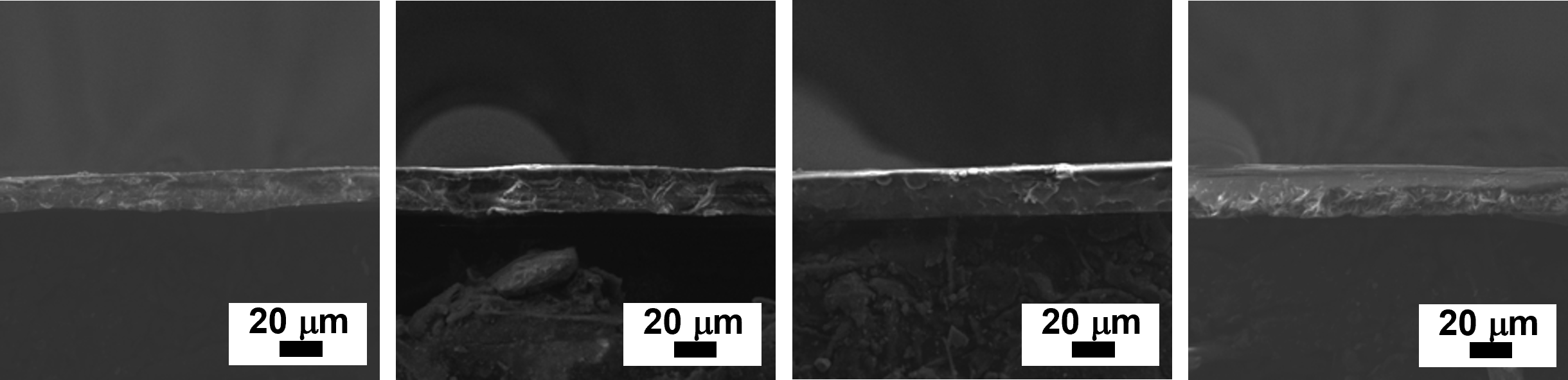
**

**Figure S9.** IR spectra of (a-e) PVP, (a) MnO_2_ NW/PVP-1~5, (b) HCMP NW/PVP-1~5, (c) MnO_2_@CMP-1/PVP-1~5, (d) MnO_2_@CMP-2/PVP-1~5, and (e) MnO_2_@CMP-3/PVP-1~5 films.

**(b)**

**(a)**


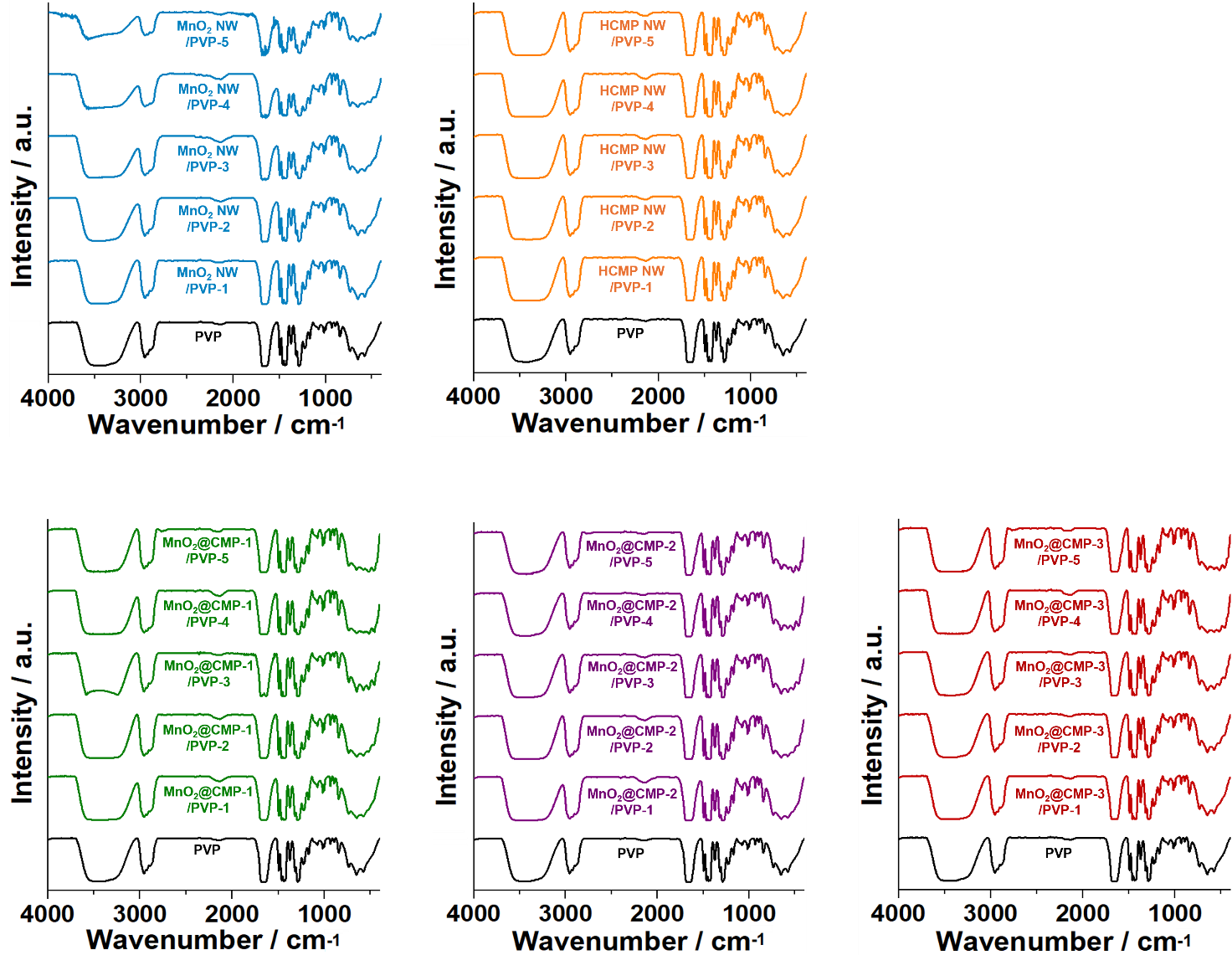


**(e)**

**(d)**

**(c)**

**Figure S10.** PXRD patterns of (a) MnO_2_ NW/PVP-1~5, (b) HCMP NW/PVP-1~5, (c) MnO_2_@CMP-1/PVP-1~5, (d) MnO_2_@CMP-2/PVP-1~5, and (e) MnO_2_@CMP-3/PVP-1~5 films.

.

**(b)**

**(a)**


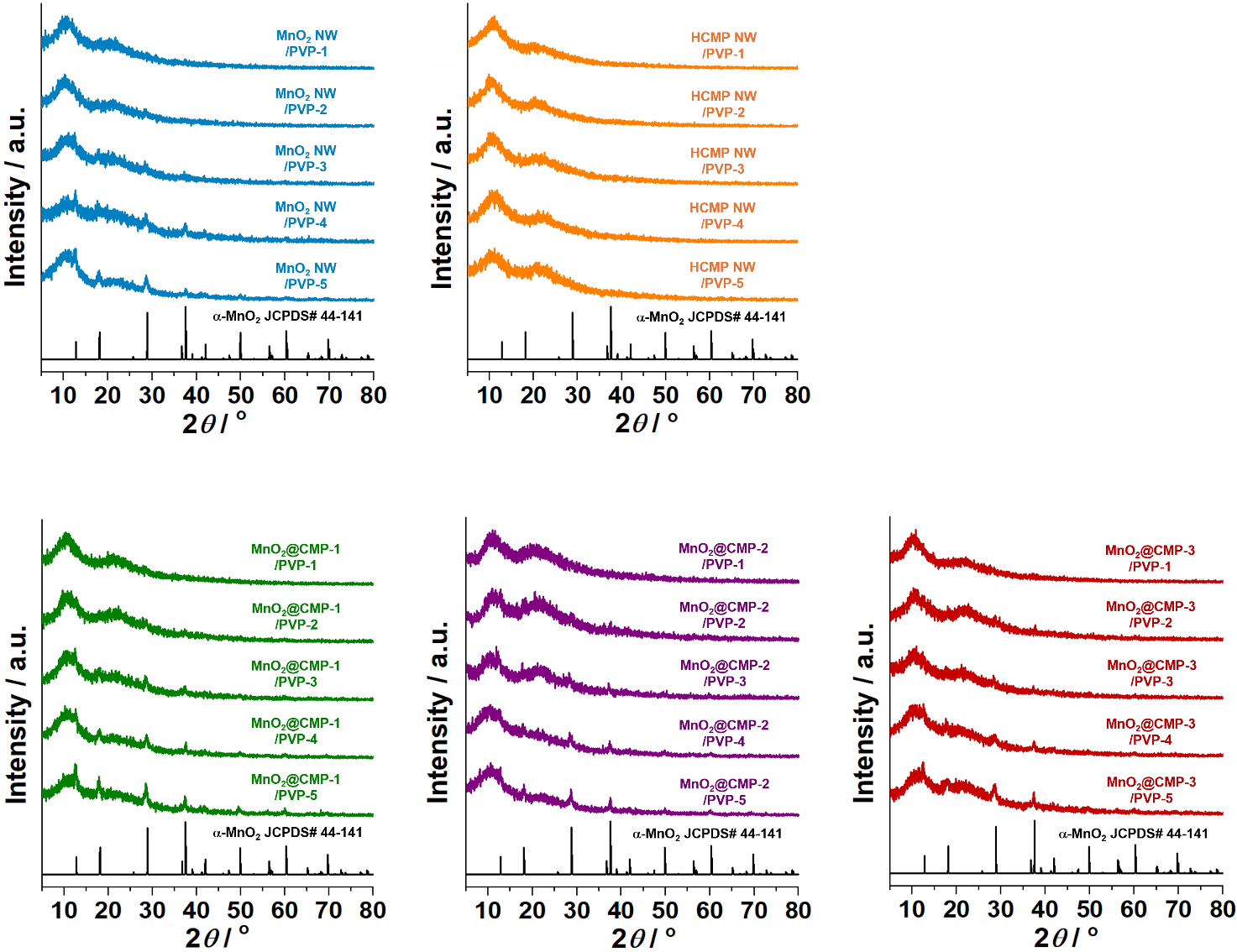


**(d)**

**(c)**

**(e)**

**Figure S11.** Output currents of (a) MnO_2_ NW/PVP-1~5, (b) HCMP NW/PVP-1~5, and (c) MnO_2_@CMP-2/PVP-1~5 films.

**(c)**

**(b)**

**(a)**


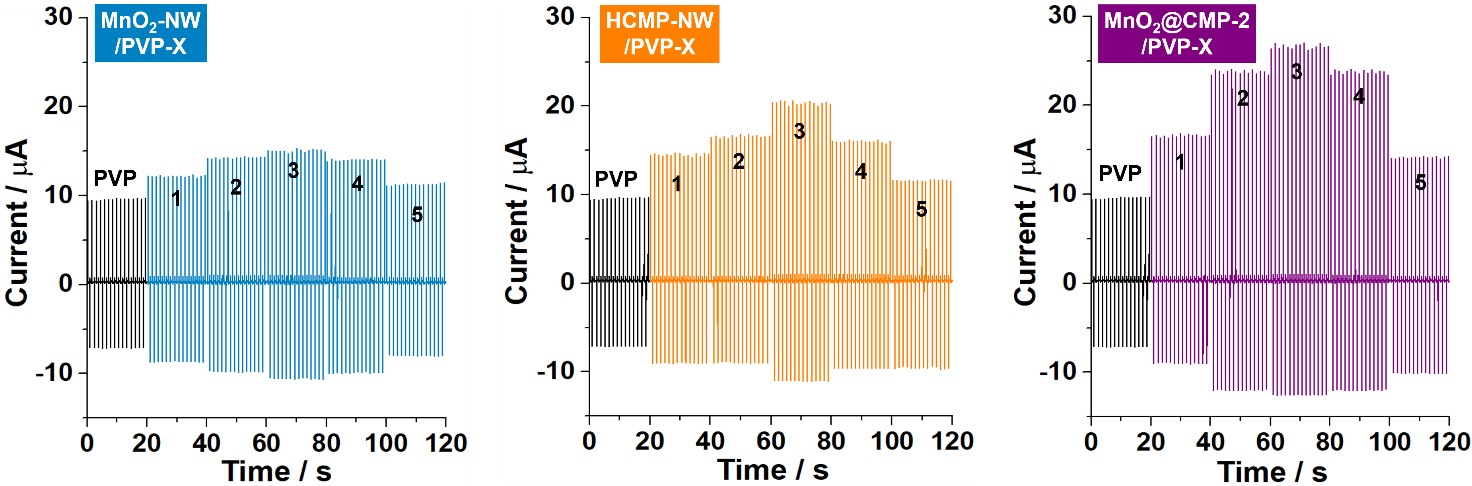


**Figure S12.** Analysis of XPS Mn 2p orbital peaks of MnO_2_ NW before and after treatment with TCNQ and TritylBF_4_.


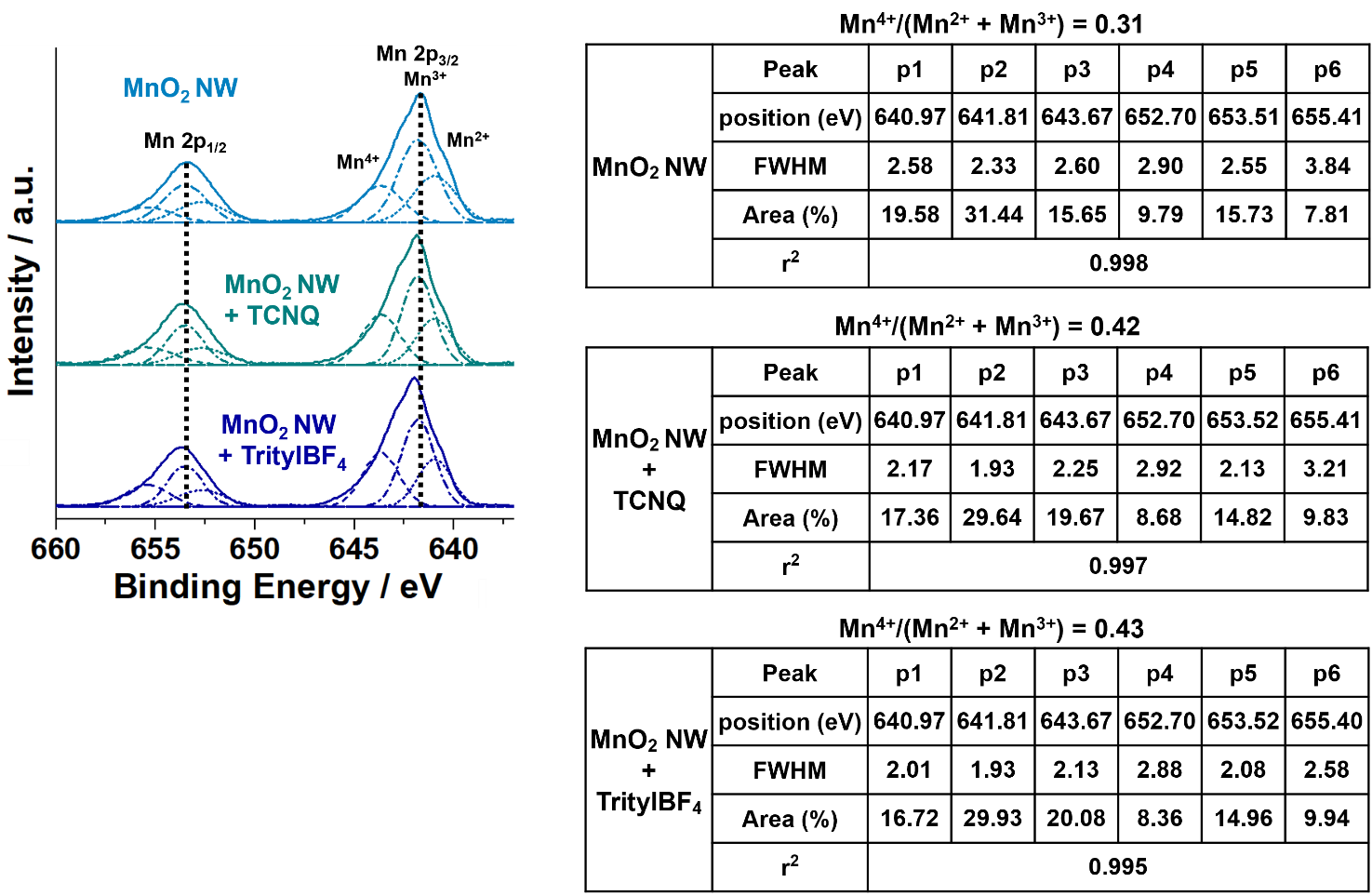


**Figure S13.** Analysis of XPS O 1s orbital peaks of MnO_2_ NW before and after treatment with TCNQ and TritylBF_4_.


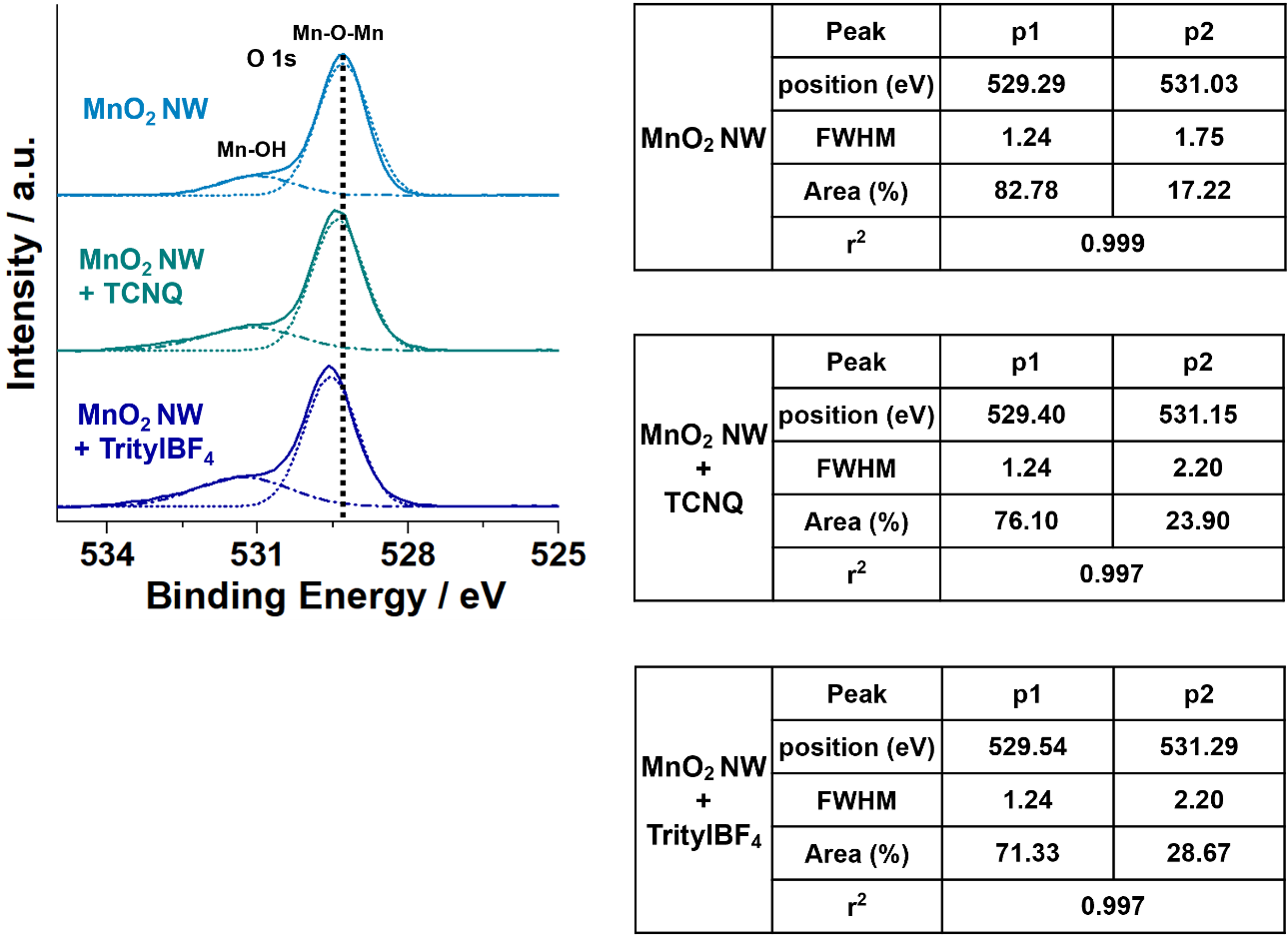


**Figure S14.** EPR spectra of the MnO_2_ NW before and after treating with TCNQ and tritylBF_4_.

**
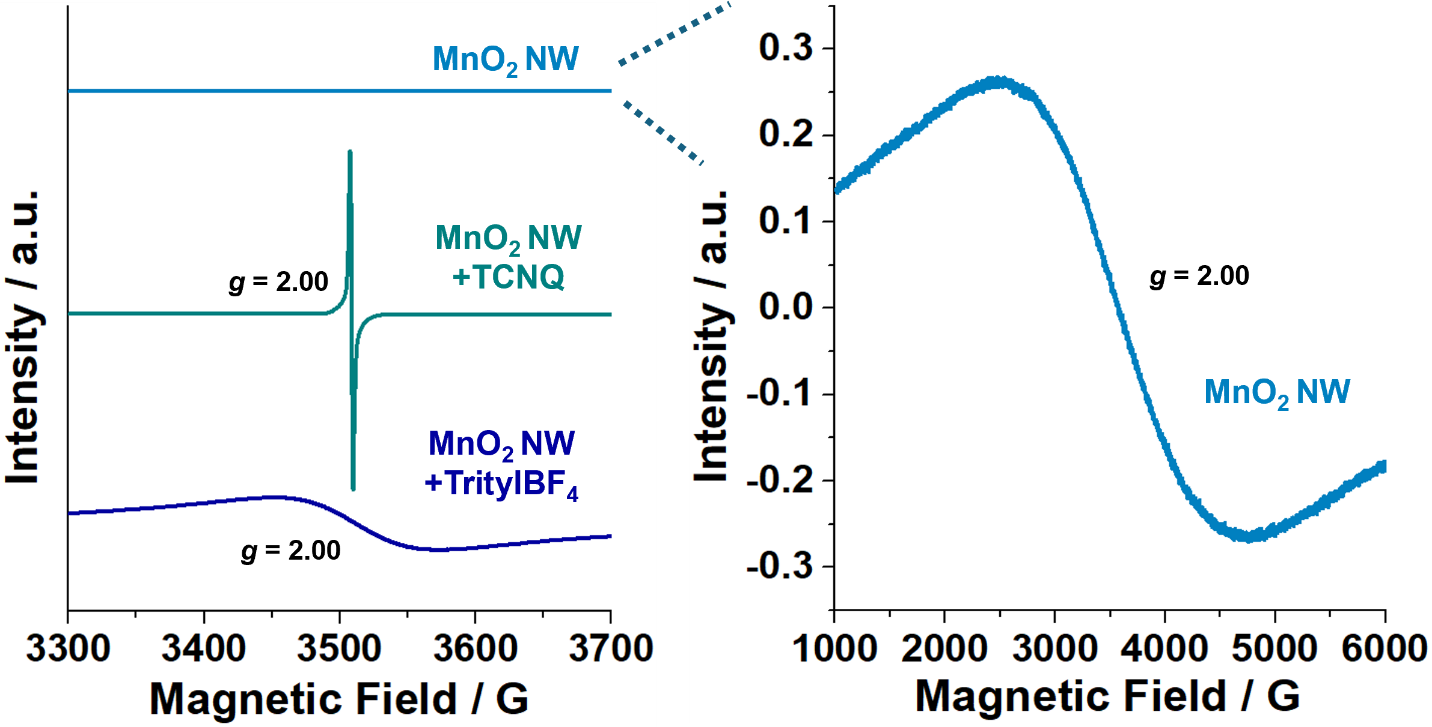
**

**Figure S15.** Triboelectric output voltages and currents of (a, d) MnO_2_@CMP-1/PVP-1~5, (b, e) MnO_2_@CMP-2/PVP-1~5, and (c, f) MnO_2_@CMP-3/PVP-1~5 films.

**(a)**

**(c)**

**(b)**


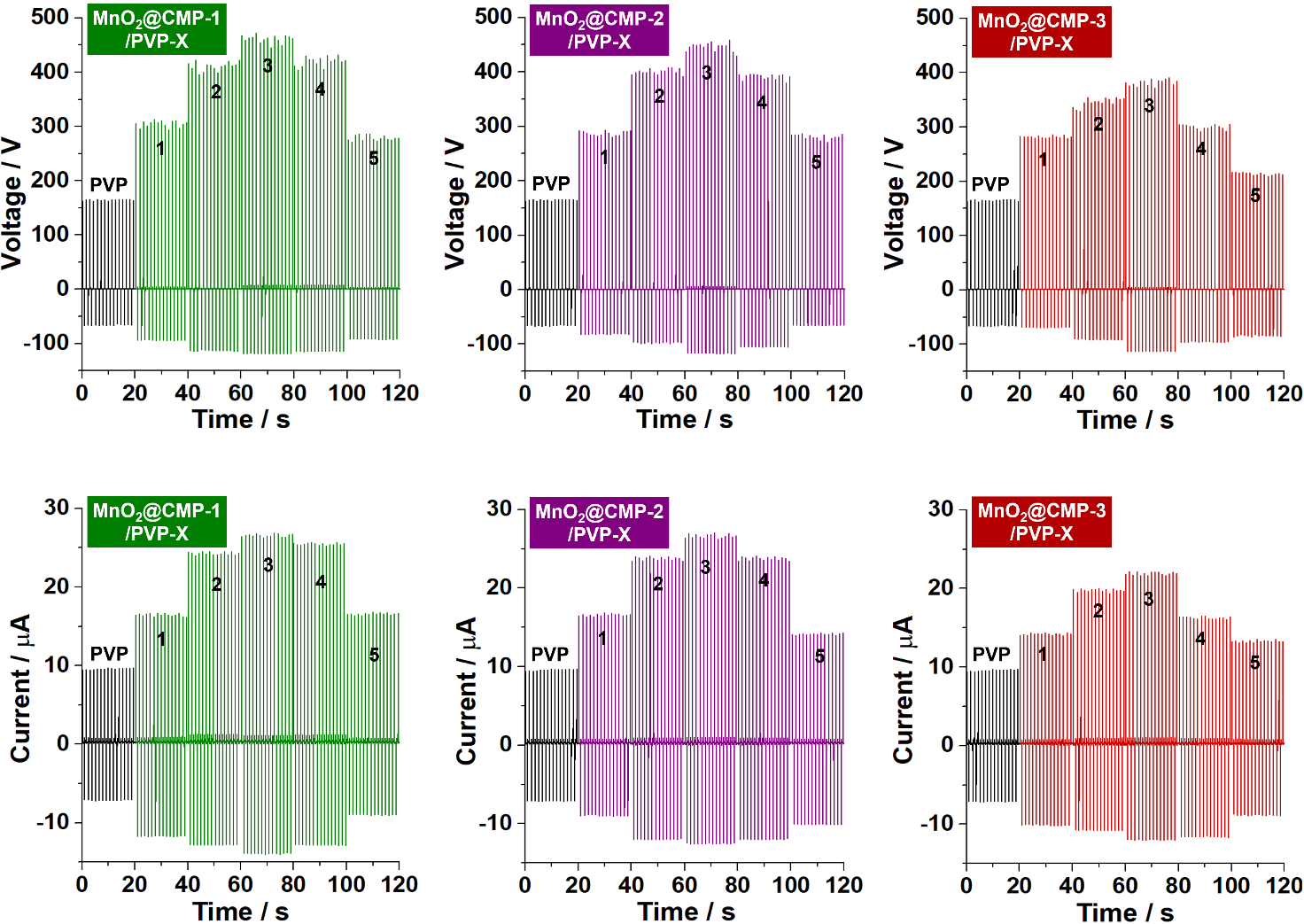


**(e)**

**(d)**

**(f)**

**Figure S16.** RH-dependent output (a) voltages and (b) currents of MnO_2_@CMP-2/PVP-3 films (working conditions: a working area of 2 cm × 2 cm, a pushing force of 2 kgf, a pushing frequency of 0.73 Hz).

**(b)**

**(a)**

**
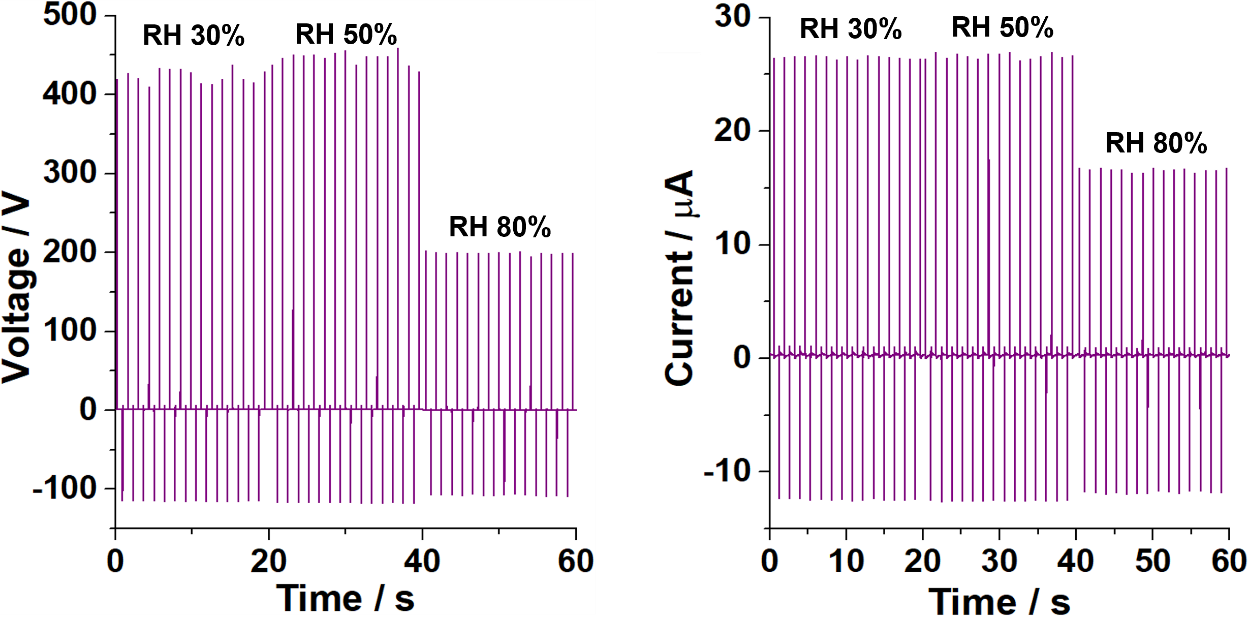
**

**Figure S17.** (a) Pushing force and (b) pushing frequency-dependent output currents of MnO_2_@CMP-2/PVP-3 films (working conditions for pushing force: a working area of 2 cm × 2 cm, a pushing frequency of 0.73 Hz, RH 50%; working conditions for pushing frequency: a working area of 2 cm × 2 cm, a pushing force of 2 kgf, RH 50%).

**(a)**

**(b)**

**
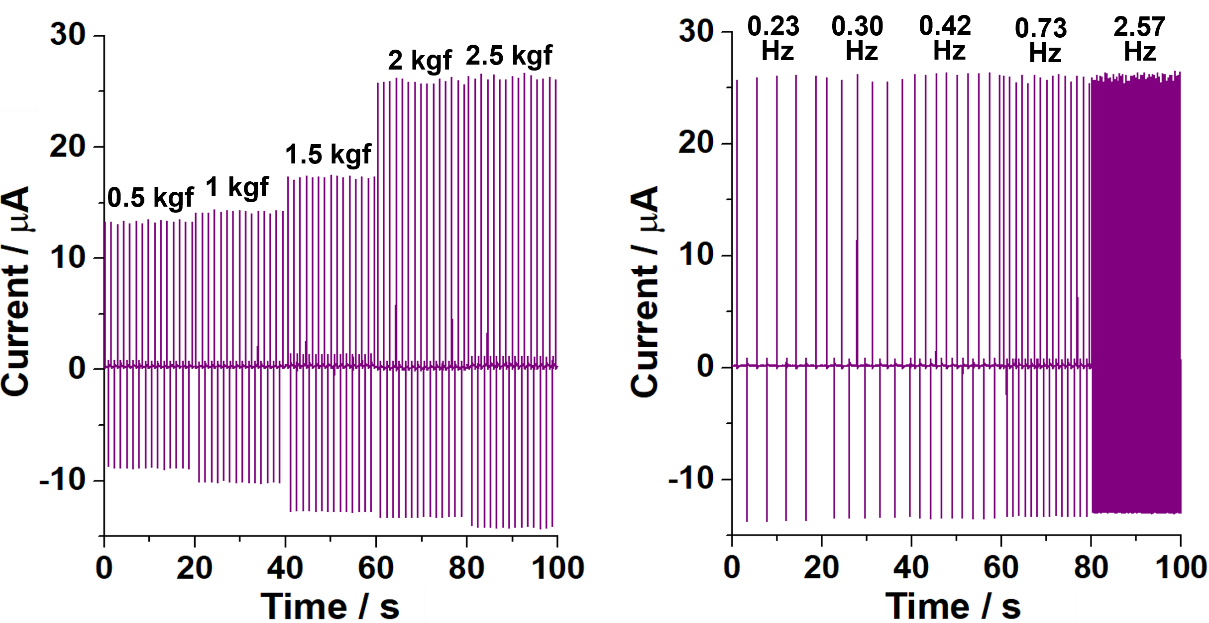
**

**Table S1.** Triboelectric performance of covalent organic framework (COF) and conjugated microporous polymer (CMP) materials in the literature.

COF: covalent organic framework. TFP-DB-COF: 1,3,5-triformylphloroglucinol-dimidium bromide-COF, PVDF: polyvinylidene fluoride, TPB-DBBA-COF: 1,3,5-tris(4-aminophenyl)benzene-2,5-dibromobenzene-1,4-dicarbaldehyde, CMP: conjugated microporous polymer, PU: polyurethane, PFA: perfluoroalkoxy alkane, ZIF: zeolitic imidazolate framework, PTFE: poly(tetrafluoroethylene), PVC: polyvinylchloride, PVA: polyvinyl alcohol, Tp-TFAB: 1,3,5-triformylphloroglucinol-1,3,5-tris(2,3,5,6-tetrafluoroaniline), CMPA: CMP aerogel, FEP: fluorinated ethylene propylene, PVP: polyvinylpyrrolidone.

[S1] L. Zhai, W. Wei, B. Ma, W. Ye, J. Wang, W. Chen, X. Yang, S. Cui, Z. Wu, C. Soutis, G. Zhu, L. Mi, *ACS Materials Lett.*, **2020**, *2*, 1691-1697.

[S2] L. Zhai, S. Cui, B. Tong, W. Chen, Z. Wu, C. Soutis, D. Jiang, G. Zhu, L. Mi, *Chem. Eur. J.,* **2020**, *26*, 5784-5788.

[S3] S. I. Park, D. -M. Lee, C. W. Kang, S. M. Lee, H. J. Kim, Y. -J. Ko, S. -W. Kim, S. U. Son, *J. Mater. Chem. A*, **2021**, *9*, 12560-12565.

[S4] C. Lin, L. Sun, X. Meng, X. Yuan, C. -X. Cui, H. Qiao, P. Chen, S. Cui, L. Zhai, L. Mi, *Angew. Chem. Int. Ed.*, **2022**, *61*, e202211601.

[S5] S. Yao, M. Zheng, S. Wang, T. Huang, Z. Wang, Y. Zhao, W. Yuan, Z. Li, Z. L. Wang, L. Li, *Adv. Funct. Mater.*, **2022**, *32*, 2209142.

[S6] S. Hajra, J. Panda, J. Swain, H. -G. Kim, M. Sahu, M. K. Rana, R. Samantaray, H. J. Kim, R. Sahu, *Nano Energy*, **2022**, *101*, 107620.

[S7] L. Shi, V. S. Kale, Z. Tian, X. Xu, Y. Lei, S. Kandambeth, Y. Wang, P. T. Parvatkar, O. Shekhah, M. Eddaoudi, H. N. Alshareef, *Adv. Funct. Mater.*, **2023**, *33*, 2212891.

[S8] N. Meng, Y. Zhang, W. Liu, Q. Chen, N. Soykeabkaew, Y. Liao, *Adv. Funct. Mater.*, **2024**, *34*, 2313534.

**Table S2.** Triboelectric performance of metal-organic framework (MOF) materials in the literature.

ZIF: zeolitic imidazolate framework, KAUST: King Abdullah University of Science and Technology, PDMS: polydimethylsiloxane, CD-MOF: cyclodextrin-MOF, PVDF: polyvinylidene fluoride, PVC: polyvinylchloride, NF-MOF: nanoflake-MOF, PTFE: poly(tetrafluoroethylene), SF: silk fibroin, CMP: conjugated microporous polymer, PVP: polyvinylpyrrolidone, PFA: perfluoroalkoxy alkane

[S9] G. Khandelwal, A. Chandrasekhar, N. P. M. J. Raj, S. -J. Kim, *Adv. Energy Mater*., **2019**, *9*, 1803581.

[S10] Y. Guo, Y. Cao, Z. Chen, R. Li, W. Gong, W. Yang, Q. Zhang, H. Wang, *Nano Energy*, **2020**, *70*, 104517.

[S11] G. Khandelwal, N. P. M. J. Raj, S. -J. Kim, *J. Mater. Chem. A*, **2020**, *8*, 17817-17825.

[S12] S. Hajra, M. Sahu, A. M. Padhan, I. S. Lee, D. K. Yi, P. Alagarsamy, S. S. Nanda, H. J. Kim, *Adv. Funct. Mater.*, **2021**, *31*, 2101829.

[S13] C. Huang, G. Lu, N. Qin, Z. Shao, D. Zhang, C. Soutis, Y. -Y. Zhang, L. Mi, H. Hou, *ACS Appl. Mater. Interfaces*, **2022**, *14*, 16424-16434.

[S14] R. A. Shaukat, Q. M. Saqib, J. Kim, H. Song, M. U. Khan, M. Y. Chougale, J. Bae, M. J. Choi, *Nano Energy*, **2022**, *96*, 107128.

[S15] Z. Chen, Y. Cao, W. Yang, L. An, H. Fan, Y. Guo, *J. Mater. Chem. A*, **2022**, *10*, 799-807.

[S16] Q. Xi, Z. Chen, Y. Li, F. Liu, Y. Guo, *ACS Appl. Electron. Mater.*, **2023**, *5*, 5215-5223
